# Bacterial specificity of the gut microbiome predicts bone density in primary hyperparathyroidism

**DOI:** 10.1038/s41413-026-00529-1

**Published:** 2026-05-25

**Authors:** Hamid Y. Dar, Jiali Fang, Sanchiti Patil, Nand K. Roy, Sanchita Agarwal, M. Neale Weitzmann, Rheinallt M. Jones, John P. Bilezikian, Roberto Pacifici

**Affiliations:** 1 Division of Endocrinology, Metabolism and Lipids, Department of Medicine, Emory University, Atlanta, GA USA; 2 Emory Microbiome Research Center, Emory University, Atlanta, GA USA; 3 Division of Endocrinology, Vagelos College of Physicians and Surgeons, Columbia University, New York, NY USA; 4 Joseph Maxwell Cleland Atlanta VA Medical Center, Decatur, GA USA; 5 Division of Pediatric Gastroenterology, Hepatology, and Nutrition, Department of Pediatrics, Emory University, Atlanta, GA USA; 6 Immunology and Molecular Pathogenesis Program, Emory University, Atlanta, GA USA

**Keywords:** Metabolic bone disease, Bone

## Abstract

Primary hyperparathyroidism causes mild-to-severe bone loss, but the reason for this heterogeneity is unclear. We investigated the role of the microbiome in 50 primary hyperparathyroidism patients. Microbiome transfers from primary hyperparathyroidism patients with and without osteoporosis to germ-free mice replicated the human bone phenotype and regulated TNF^+^ T cells and Th17 cells in mice. Accordingly, circulating TNF^+^ T cells and Th17 cells and TNF/IL17 production predicted bone density in primary hyperparathyroidism patients. *Bifidobacterium longum*, TNF^+^ T cells, and Th17 cells were mediators of bone loss in primary hyperparathyroidism patients, while *Bifidobacterium longum* supplementation caused PTH to expand TNF^+^ T cells and Th17 cells and induce bone loss in mice. Our findings link *Bifidobacterium longum*-induced TNF^+^ T cells and Th17 cells to bone loss in patients with primary hyperparathyroidism. *Bifidobacterium longum* abundance may determine the skeletal phenotypes of patients with primary hyperparathyroidism and allow prediction of their risk of bone loss. Microbiome modifications by antibiotics or precision probiotics might offer novel preventive approaches for the skeletal complications of primary hyperparathyroidism.

## Introduction

Primary hyperparathyroidism is a common endocrine disorder, mainly affecting post-menopausal women.^1^ It arises from overactive parathyroid tissue, typically a single adenoma or multiglandular hyperplasia, leading to excessive PTH secretion.^2^ This increases bone loss and fracture risk in some, but not all patients.^2–4^ The mechanisms driving this heterogeneity are unknown, and no reliable predictive biomarker exists.

Animal studies have revealed that PTH binding to the type 1 PTH/PTHrP receptor (PTHR1), expressed by osteoblasts and osteocytes, contributes to PTH-associated bone loss.^5,6^ Also noteworthy is the observation that the PTHR1 receptor is expressed by T cells.^7,8^ PTH binding to T cells is required for PTH to stimulate bone resorption^9^ and bone formation,^10^ and cause bone loss in mice.^9,10^ Accordingly, continuous PTH (cPTH) infusion in mice, a model of primary hyperparathyroidism, fails to cause trabecular bone loss in T cell-deficient mice^11–14^ and in mice selectively lacking PTHR1 expression by T cells.^9^

Studies in pre-clinical models have also established the contribution of the gut microbiome as a mediator of the effects of PTH in bone.^14–16^ In fact, both germ-free mice and conventional mice treated with broad-spectrum antibiotics to deplete the microbiome are resistant to the effects of PTH on bone turnover and trabecular bone microarchitecture.^14,15^ Mechanistically, gut bacteria and bacterial products induce intestinal TNFα (TNF) producing T cells (TNF^+^ T cells) that migrate to the bone marrow (BM) and release TNF in response to PTH.^14^ Specific bacteria also activate and expand intestinal IL-17-producing Th17 cells, which migrate to the BM in response to the chemokine ligand CCL20 produced by BM cells and induced by TNF.^14^ CCL20 is the ligand for the chemokine receptor CCR6, which is expressed by Th17 cells.^17^ Attesting to the functional relevance of the CCR6/CCL20 axis, blockade of CCL20 by anti CCL20 antibody prevents PTH-induced bone loss.^14^ Th17 cell-produced IL-17 stimulates osteocytic RANKL expression,^13^ while TNF potentiates the effects of RANKL,^18^ a factor that also induces osteoclastogenesis and bone loss.^13^ Although the role of TNF in humans affected by primary hyperparathyroidism remains to be investigated, primary hyperparathyroidism increases the production of IL-17 by peripheral blood mononuclear cells (PBMCs), a phenomenon that can be reversed by parathyroidectomy.^12^

In mice, intestinal TNF^+^ T cells are induced by a variety of bacteria and bacterial products.^19–22^ By contrast, Th17 cells form in the gut primarily in response to segmented filamentous bacteria (SFB), which are spore-forming, Gram-positive commensal bacteria.^23–25^ Accordingly, mice lacking SFB in their microbiome are protected against the bone-wasting activity of PTH, while those with SFB in their microbiome respond to PTH with an expansion of Th17 cells and bone loss.^14^ Over 20 non-virulent gut bacterial strains have been identified that induce Th17 cell differentiation in the human gut.^26,27^ Because of the significant heterogeneity in gut microbiome composition among populations, including considerable variation in the frequency or presence of bacteria that activate TNF^+^ T cells and Th17 cell differentiation, it seems plausible that the capacity of primary hyperparathyroidism to induce skeletal manifestations is dependent on the relative abundance of bacterial species that induce TNF^+^ T cells and/or Th17 cell differentiation and expansion.

In the current study, we translated our previous pre-clinical studies into humans by analyzing the microbiome composition and the bone structure of subjects with primary hyperparathyroidism and further validated the mechanistic link underlying these clinical outcomes using mouse models. We show that the extent to which primary hyperparathyroidism impacts the human skeleton correlated with the abundance of *Bifidobacterium longum*, a taxon that we show to induce the expansion of both intestinal and BM TNF^+^ T cells and Th17 cells.

We also show that the effects of primary hyperparathyroidism on the human skeleton were correlated with the number and activity of TNF^+^ T cells and Th17 cells. Our data implicate T cell-produced TNF and IL-17 in the bone loss induced by primary hyperparathyroidism and suggest that the composition of the intestinal microbiome can predict the propensity of patients with primary hyperparathyroidism to develop bone loss and osteoporosis.

## Results

### The gut microbiome of primary hyperparathyroidism patients is a transmissible regulator of bone density

**Fig. 1:**
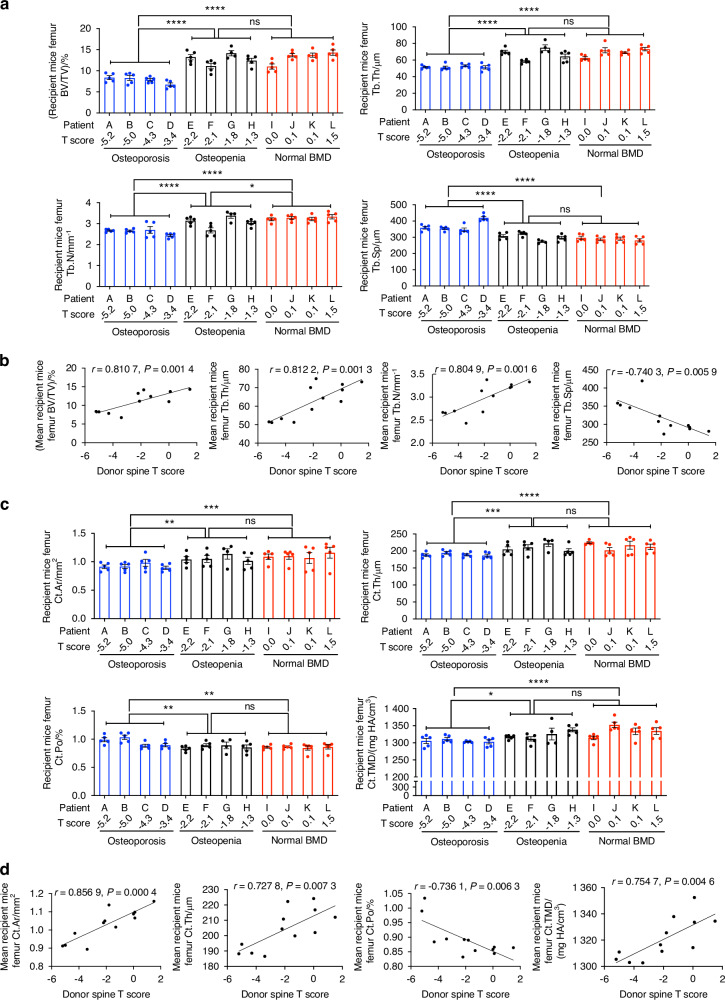
Indices of femoral bone structure in germ-free mice with microbiome from primary hyperparathyroidism patients with spine osteoporosis, osteopenia, or normal BMD. The measured indices were trabecular bone volume fraction (BV/TV), trabecular thickness (Tb.Th), trabecular number (Tb.N), trabecular separation (Tb.Sp), cortical area (Ct.Ar), cortical thickness (Ct.Th), cortical porosity (Ct.Po), and cortical tissue mineral density (Ct.TMD). In (**a**, **c**), each dot represents a recipient mouse. Each letter represents a patient. Mice were divided into three groups based on the spine T score of the human donors, and the groups were compared by one-way ANOVA and post hoc tests applying Bonferroni’s correction for multiple comparisons. Data are shown as mean ± SEM. *n* = 4–5 recipient mice for each human donor. In (**b**, **d**), the spine T score of each human donor was plotted against the mean of the values measured in the 4–5 recipient mice with the microbiome from the same donor. *r* and *P* values were calculated using Pearson correlations. The curves show simple linear regression lines. *P < 0.05, **P < 0.01, ***P < 0.001, and ****P < 0.000 1

50 primary patients with hyperparathyroidism were enrolled in this study. Demographic data and clinical parameters are described in Table 1. At the time of enrollment, stool samples were collected and analyzed by metagenomic sequencing using established methods.^28^ Areal BMD was measured by dual X-ray absorptiometry (DXA) at the 1/3 radius, lumbar spine, total hip, and femoral neck (FN). Volumetric and structural indices of cortical and trabecular bone were measured at the radius and tibia by high-resolution peripheral quantitative computed tomography (HRpQCT). Circulating TNF^+^ T cells and Th17 cells were enumerated by flow cytometry following the gating strategy outlined in supplementary Fig. 1a. To assess the impact of the gut microbiome on the bone loss induced by primary hyperparathyroidism, patients with primary hyperparathyroidism meeting clinical criteria of osteoporosis, osteopenia, or normal BMD at the lumbar spine and 1/3 radius were randomly selected (*n* = 4 patients per group) from the entire cohort and utilized as stool donors in fecal material transfer (FMT) experiments. Demographic data and clinical characteristics of these 12 donors are described in (Table 2). Representative flow cytometry data from 1 patient per group are shown in Supplementary Fig. 1b, c. Stool liquid suspensions from these 12 donors were transferred by oral gavage into 4-week-old recipient germ-free mice (*n* = 4–5 mice per stool donor). Recipient mice were fed a standard diet until 16 weeks of age, and then a low-calcium diet for 4 weeks, an intervention that elevates circulating PTH levels and induces bone loss,^14,29^ similarly to cPTH infusion,^14,29^ a procedure that is technically challenging to perform in germ-free mice. Recipient mice were housed in a Tecniplast ISOcageP Bioexclusion system^30^ to prevent alterations of the microbiome composition by environmental bacteria. To investigate if BMD-defining elements of the microbiome originating from human donors had successfully transferred in recipient germ-free mice, stool samples were collected from mice at the end of the experiments, and the gut microbiome composition was analyzed at the phylum and species level (Supplementary Fig. 2a, b). Analysis at the species level revealed significant differences in α diversity and β diversity in recipient mice with microbiomes originating from donors with osteoporosis, osteopenia, or normal BMD (Supplementary Fig. 2c, d). μCT analysis of mouse femurs harvested at the end of the experiment revealed that mice with microbiome from subjects with primary hyperparathyroidism and osteoporosis had lower femoral trabecular bone volume fraction (BV/TV), lower trabecular thickness (Tb.Th) and trabecular number (Tb.N), and higher trabecular separation (Tb.Sp) than mice with microbiome from patients with primary hyperparathyroidism and osteopenia or normal BMD (Fig. 1a and Fig. S3a). Across the groups, T scores at the spine and radius of the human stool donors directly correlated with femoral BV/TV and indices of trabecular structure of recipient mice (Fig. 1b and Fig. S3b).

**Table 1:** Demographic and clinical characteristics (mean ± SD) of the patients at baseline (*n* = 50)

Characteristic	
Age/year	62.18 ± 10.74
Sex, no. (%)	
Male	9 (18.00%)
Female	41 (82.00%)
Race, no. (%)	
White	41 (82.00%)
Black or African American	2 (4.00%)
Asian	2 (4.00%)
More than one race	0
Other	5 (10.00%)
Ethnic group, no. (%)	
Hispanic	7 (14.00%)
Non-Hispanic	42 (84.00%)
Unknown	1 (2.00%)
Body-mass index^a^	24.93 ± 4.06
Bone mineral density T scores	
Lumbar spine	−1.77 ± 1.54
Total hip	−1.55 ± 1.11
Femoral neck	−1.90 ± 1.13
Distal 1/3 radius	−1.72 ± 1.56
Osteoporosis, no. (%)	15 (30.00%)
Osteopenia, no. (%)	18 (36.00%)
Normal bone density, no. (%)	17 (34.00%)
History of fracture, no. (%)^b^	
Yes	31 (62.00%)
No	19 (38.00%)
Screening lab values^c^	
Serum calcium (8.6–10.4 mg/dL)	10.61 ± 0.71
Serum PTH (15.1–85.7 pg/mL)	78.12 ± 31.18
Serum 25OHD (>20 ng/mL)	34.53 ± 12.57
Serum CTX (0.14–1.35 ng/mL)	0.80 ± 0.46
Serum P1NP/(ng/mL)	71.00 ± 33.56

^a^The body-mass index is the weight in kilograms divided by the square of the height in meters

^b^Excludes fractures of the skull, face, fingers, and toes, which are typically not associated with osteoporosis, as well as fractures associated with high trauma severity

^c^Numbers in parentheses are the reference range

**Table 2:** Demographic and clinical characteristics (mean ± SD) of the stool donor patients utilized for FMT studies

Characteristic	Normal BMD	Osteopenia	Osteoporosis
Age/year	62.50 ± 10.02	57.25 ± 7.93	69.75 ± 8.57
Sex, no. (%)			
Male	1 (25.00%)	0	0
Female	3 (75.00%)	4 (100.00%)	4 (100.00%)
Race, no. (%)			
White	4 (100.00%)	2 (50.00%)	3 (75.00%)
Black or African American	0	1 (25.00%)	0
Asian	0	0	1 (25.00%)
More than one race	0	0	0
Other	0	1 (25.00%)	0
Ethnic group, no. (%)			
Hispanic	0	1 (25.00%)	0
Non-Hispanic	4 (100.00%)	3 (75.00%)	4 (100.00%)
Unknown	0	0	0
Body-mass index^a^	25.68 ± 4.91	24.35 ± 4.40	20.83 ± 2.07
Bone mineral density T scores			
Lumbar spine	0.42 ± 0.71	−1.85 ± 0.40**	−4.47 ± 0.81****
Total hip	−0.52 ± 0.28	−1.27 ± 0.70	−2.95 ± 1.19**
Femoral neck	−0.82 ± 0.92	−1.65 ± 0.65	−3.15 ± 0.91*
Distal 1/3 radius	−0.72 ± 1.10	−0.80 ± 1.46*	−3.90 ± 0.57**
History of fracture, no. (%)^b^	3 (75.00%)	2 (50.00%)	1 (25.00%)
Screening lab values^c^			
Serum calcium (8.6–10.4 mg/dL)	10.33 ± 0.97	11.08 ± 0.82	11.30 ± 0.86
Serum PTH (15.1–85.7 pg/mL)	96.23 ± 11.89	71.83 ± 12.47	107.1 ± 56.88
Serum 25OHD (>20 ng/mL)	24.29 ± 7.91	35.56 ± 13.98	30.52 ± 10.77
Serum CTX (0.14–1.35 ng/mL)	0.87 ± 0.56	0.79 ± 0.34	1.23 ± 1.00
Serum P1NP/(μg/L)	93.05 ± 51.80	92.05 ± 28.49	87.03 ± 33.54

*P < 0.05, **P < 0.01, and ****P < 0.001 compared to normal BMD group

^a^The body-mass index is the weight in kilograms divided by the square of the height in meters

^b^Excludes fractures of the skull, face, fingers, and toes, which are typically not associated with osteoporosis, as well as fractures associated with high trauma severity

^c^Numbers in parentheses are the reference range

**Fig. 2:**
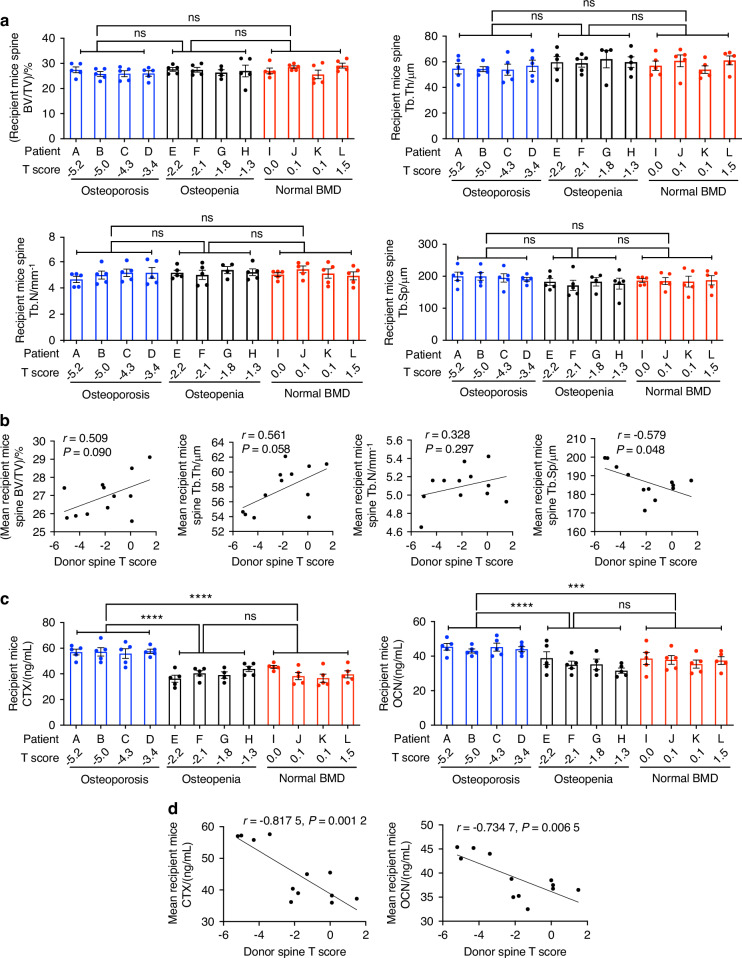
Indices of spinal bone structure and of bone turnover in germ-free mice with microbiome from primary hyperparathyroidism patients with spine osteoporosis, osteopenia, or normal BMD. The measured indices were trabecular bone volume fraction (BV/TV), trabecular thickness (Tb.Th), trabecular number (Tb.N), trabecular separation (Tb.Sp), C-terminal telopeptide (CTX), which is a marker of bone resorption, and osteocalcin (OCN), which is a marker of bone formation. In (**a**, **c**), each dot represents a recipient mouse. Each letter represents a patient. Mice were divided into three groups based on the spine T score of the human donors, and the groups were compared by one-way ANOVA and post hoc tests applying Bonferroni’s correction for multiple comparisons. Data are shown as mean ± SEM. *n* = 4–5 recipient mice for each human donor. In (**b**, **d**), the spine T score of each human donor was plotted against the mean of the values measured in the 4–5 recipient mice with the microbiome from the same donor. *r* and *P* values were calculated using Pearson correlations. The curves show simple linear regression lines. *P < 0.05, **P < 0.01, ***P < 0.001, and ****P < 0.000 1

Femoral cortical area (Ct.Ar), cortical thickness (Ct.Th), cortical porosity (Ct.Po), and cortical total mineral density (Ct.TMD) were also lower in mice with microbiome from subjects with primary hyperparathyroidism and osteoporosis as compared to those with microbiome from patients with primary hyperparathyroidism and osteopenia or normal BMD (Fig. 1c and Fig. S3c). Moreover, T scores at the spine and radius of the human stool donors directly correlated with femoral Ct.Ar, Ct.Th, Ct.Po, and Ct.TMD of recipient mice (Fig. 1d and Fig. S3d). By contrast, all mice had similar indices of spinal trabecular volume and structure, regardless of the BMD of the human donors (Fig. 2a and Supplementary Fig. 3e). Moreover, T scores at the spine and 1/3 radius of the human stool donors were not significantly correlated with spinal BV/TV and indices of trabecular structure of recipient mice (Fig. 2b and Fig. S3f).

These findings demonstrated that the human donor bacteria that successfully engrafted in mice directly induced femoral trabecular bone loss but not spinal bone loss in recipient mice. Analysis of biochemical markers of bone turnover revealed that mice with microbiome from subjects with primary hyperparathyroidism and osteoporosis had higher serum levels of C-terminal telopeptide (CTX) and osteocalcin (OCN), which are markers of bone resorption and bone formation respectively (Fig. 2c and Fig. S3g). Moreover, T scores at the spine and radius of the human stool donors were inversely correlated with CTX and OCN (Fig. 2d and Fig. S3h). These findings demonstrated that the human gut microbiomes directly induced femoral bone loss and regulated bone turnover in recipient mice, without causing spinal bone loss.

**Fig. 3:**
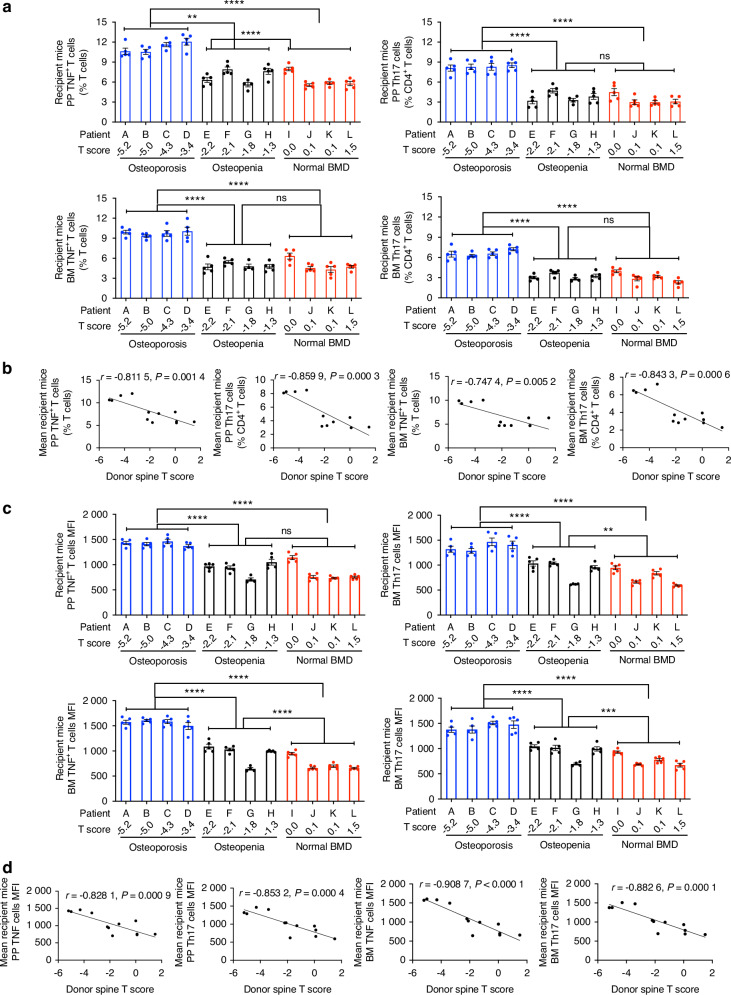
Frequency and MFI of PP and BM of TNF^+^ T cells and Th17 cells in WT germ-free mice with microbiome from primary hyperparathyroidism patients with spine osteoporosis, osteopenia, or normal BMD. In (**a**, **b**), each dot represents a recipient mouse. Each letter represents a patient. Mice were divided into three groups based on the spine T score of the human donors, and the groups were compared by one-way ANOVA and post hoc tests applying Bonferroni’s correction for multiple comparisons. In (**c**, **d**), the spine T score of each human donor was plotted against the mean of the values measured in the recipient mice with the microbiome from the same donor. *r* and *P* values were calculated using Pearson correlations. The curves show simple linear regression lines. All data were normally distributed. Data are shown as mean ± SEM. *n* = 4–5 WT mice per human donor. *P < 0.05, **P < 0.01, ***P < 0.001, and ****P < 0.000 1. ns not significant

Microbiomes from osteoporotic subjects induced higher frequencies of Peyer’s patches (PPs) and BM TNF^+^ T cells and Th17 cells in recipient mice than those from non-osteoporotic subjects (Fig. 3a and Fig. S4a). Inverse correlations were also detected between T scores at the spine and radius of the primary hyperparathyroidism subjects and the frequency of PP and BM TNF^+^ T cells and Th17 cells in recipient mice (Fig. 3b and Fig. S4b). The mean fluorescence intensity (MFI) of PP and BM TNF^+^ T cells and Th17 cells, which is a quantitative measure of the mean production/cell of TNF and IL-17 proteins, was higher in recipient mice with microbiomes from osteoporotic subjects (Fig. 3c and Fig. S4c), and T scores of subjects with primary hyperparathyroidism were inversely correlated with MFI of PP and BM TNF^+^ T cells and Th17 cells in recipient mice (Fig. 3d and Fig. S4d). These findings demonstrated that the human gut microbiomes directly induced bone loss and expanded TNF and IL-17 producing T cells in recipient mice.

### The gut microbiome of primary hyperparathyroidism patients regulates the migration of intestinal TNF^+^ T cells and Th17 cells to the BM

**Fig. 4:**
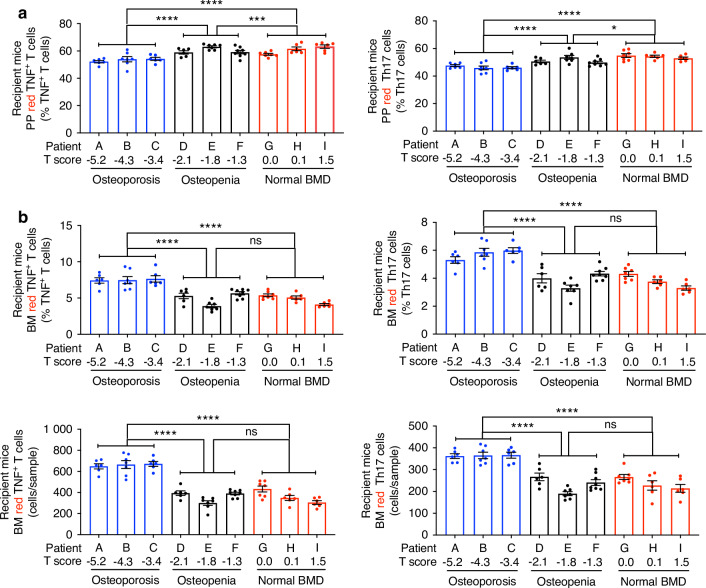
Frequency of red fluorescent-tagged TNF^+^ T cells and Th17 cells in germ-free Kaede mice with microbiome from primary hyperparathyroidism patients with spine osteoporosis, osteopenia, or normal BMD. In (**a**, **b**), each dot represents a recipient mouse. Each letter represents a patient. Mice were divided into three groups based on the spine T score of the human donors, and the groups were compared by one-way ANOVA and post hoc tests applying Bonferroni’s correction for multiple comparisons. All data were normally distributed. Data are shown as mean ± SEM. *n* = 6–8 Kaede mice per human donor. *P < 0.05, **P < 0.01, ***P < 0.001, and ****P < 0.000 1. ns not significant

TNF^+^ T cells and Th17 cells induce bone loss by secreting osteoclastogenic cytokines in the BM microenvironment,^14^ implying that intestinal T cells need to migrate to the BM to induce bone loss. To investigate whether primary hyperparathyroidism promotes the migration of intestinal T cells to the BM via a microbiome-dependent mechanism, we used oral gavage of liquid stool suspensions to transfer the microbiome of subjects with primary hyperparathyroidism into 4-week-old germ-free Kaede mice.^31^ These mice express a photoconvertible protein that permanently changes its emission from green to red upon photoactivation by near-UV light. The photoconversion of intracellular Kaede does not affect the cellular function and homing capacity of T cells.^32^ Thus, photoconverting cells (hereafter referred to as KaedeR cells) exclusively in the gut, and enumerating KaedeR in the BM by flow cytometry, is a sensitive method of analyzing gut-to-BM cell trafficking.^32,33^ Four-week-old recipient mice were fed a standard diet until 16 weeks of age, and then a low-calcium diet for 4 weeks to increase PTH levels. All animals were then laparotomized, and 4 PPs per mouse were photoactivated. Mice were sacrificed 24 h later, and the number of KaedeR TNF^+^ T cells and Th17 cells in PPs and BM were measured by flow cytometry. Since calculations of the absolute number of PP cells are inaccurate due to the variable size of the collected PP tissue, PP KaedeR T cells were quantified only as percentage of total cells. Analysis detected lower frequencies of PP KaedeR TNF^+^ T cells and Th17 cells, and higher frequencies of BM KaedeR TNF^+^ T cells and Th17 cells in recipient mice with a microbiome from osteoporotic patients, compared to recipient mice with a microbiome from subjects with osteopenia and/or normal BMD (Fig. 4a, b and Fig. S4e, f). These data demonstrate that the microbiome of subjects with primary hyperparathyroidism and osteoporosis promoted a higher rate of migration of TNF^+^ T cells and Th17 cells from the intestine to the BM.

### Circulating TNF^+^ T cells and Th17 cells predict bone density and bone structure in subjects with primary hyperparathyroidism

**Fig. 5:**
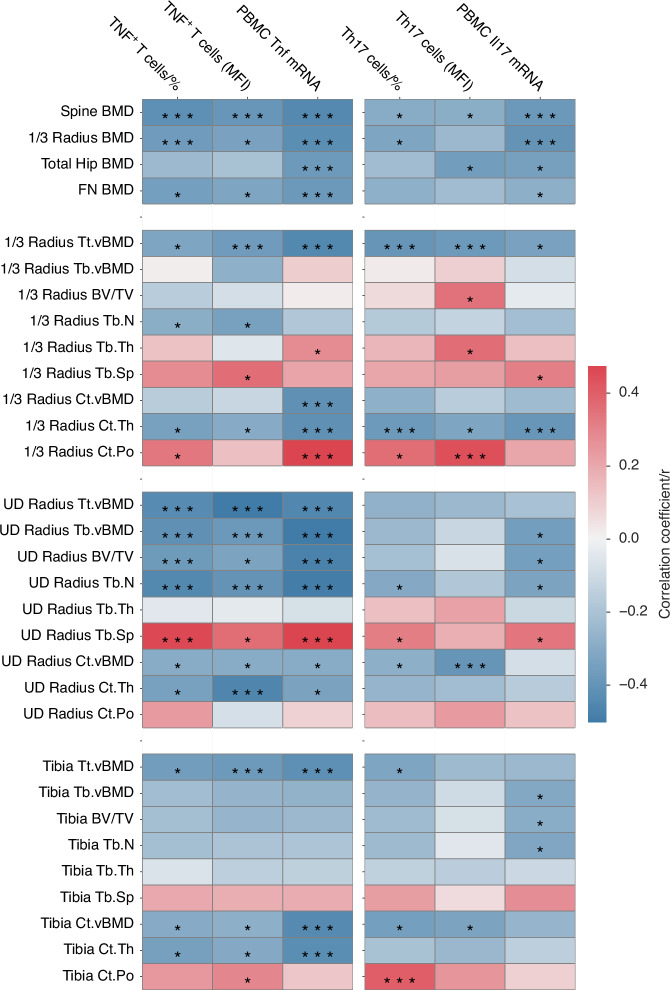
Correlations between frequency and MFI of circulating TNF^+^ T cells and Th17 cells, PBMC *Tnf* and *Il17* transcripts versus bone indices measured by DXA and HRpQCT. Bone mineral density (BMD), femoral neck (FN), ultra distal (UD), total volumetric BMD (Tt.vBMD), trabecular volumetric BMD (Tb.vBMD), cortical volumetric BMD (Ct.vBMD), bone volume fraction (BV/TV), trabecular number (Tb.N), trabecular thickness (Tb.Th), trabecular separation (Tb.Sp), cortical thickness (Ct.Th), cortical porosity (Ct.Po). The r value and the color gradient refer to Pearson’s correlation coefficient. *P < 0.05, **P < 0.01, and ***P < 0.001. Non-significant correlations are not labeled. *n* = 49 for total hip and FN BMD. *n* = 50 for all other variables. DXA of the proximal femur was not done in one patient due to hip replacements

The finding that the gut microbiome of patients with primary hyperparathyroidism directly regulates the expansion of TNF^+^ T cells and Th17 cells and the bone volume of recipient mice indicates that TNF^+^ T cells and Th17 cells and their signature cytokines play a causal role in primary hyperparathyroidism-induced bone loss. To further investigate the role of TNF and IL-17 produced by T cells in inducing bone loss in patients with primary hyperparathyroidism, we correlated bone indices to TNF^+^ T cells and Th17 cells, and *Tnf* and *Il17* cytokine transcript levels in PBMC (Fig. 5, Table S1, and Fig. S5a–f). We also computed the same correlations in the 3 subsets of patients with normal BMD, osteopenia, or osteoporosis (Table S2). In the entire population, all indices of T cell production of TNF and IL-17 were inversely correlated with and significant predictors of spine BMD. Most indices of T cell TNF and IL-17 production were also inversely correlated with 1/3 radius BMD. Cytokine transcript levels of *Il17* and Th17 cell MFI were correlated with total hip BMD, while FN BMD was predicted by *Tnf* and *Il17* transcripts, and TNF^+^ T cell frequency and MFI.

HRpQCT measurements at the 1/3 radius, a site rich in cortical bone, revealed that total volumetric BMD (Tt.vBMD), Ct.Th were inversely related and Ct.Po is positively associated with most indices of T cell TNF and IL-17 production (Fig. 5 and Table S1). T cell-derived TNF and IL-17 were poor predictors of trabecular volume and structure at this site. Measurements at the UD radius, a site richer in trabecular bone, showed that all indices of T cell TNF production were inversely correlated with total, cortical, and trabecular volumetric BMD, as well as with BV/TV, Tb.N, and Tb.Sp (Fig. 5 and Table S1). By contrast, Th17 cells and their production of IL-17 were weaker predictors of trabecular and cortical volume and structure at this site. Similarly, HRpQCT measurements at the tibia revealed strong inverse correlations between indices of T cell TNF production and total and cortical volume and Ct.Th, whereas Th17 cells and their production of IL-17 could predict several indices of trabecular and cortical volumetric BMD and Ct.Po (Fig. 5 and Table S1). These findings highlighted the relevance of TNF and IL-17 produced by T cells for the bone loss induced by primary hyperparathyroidism.

When the subsets of patients with normal BMD, osteopenia or osteoporosis were analyzed separately, correlation trends were still found, but most correlations were not significant due to the smaller sample size (Table S2).

### Microbiome-induced TNF^+^ T cells and Th17 cells mediate the effects of primary hyperparathyroidism in humans and are sufficient for PTH to cause bone loss in mice

**Fig. 6:**
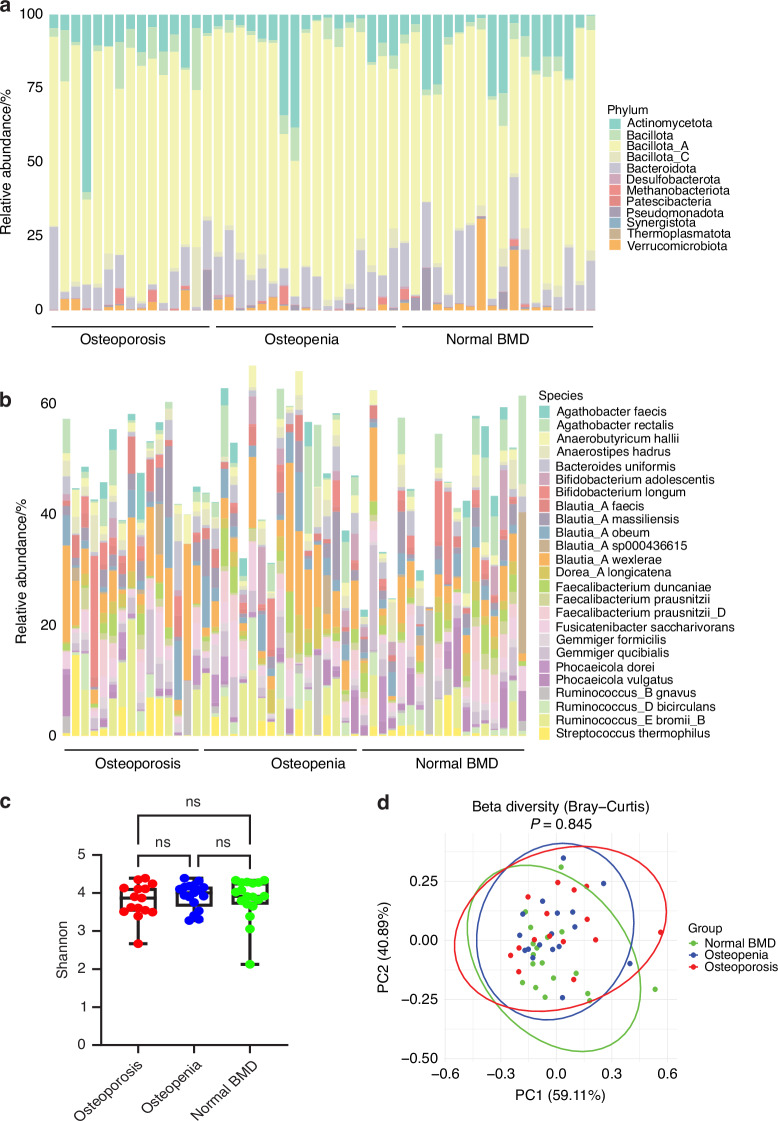
Analysis of stool microbiome in primary hyperparathyroidism patients with osteoporosis, osteopenia, or normal bone density. **a** Relative phylum level abundance. **b** Relative species level abundance of the 25 most abundant species. **c** Bacterial species alpha diversity analysis (Shannon diversity index). Kruskal–Wallis test was used to test for significant differences between groups. The box shows the median and Q1–Q3 interquartile range, and the bars show the minimum and maximum values. **d** Principal coordination analysis (PCoA) of bacterial species based on the Bray-Curtis distance metrics. PERMANOVA multivariate analysis was used to test for significant differences between groups. *n* = 15–18 patients per group. ns not significant

To identify gut microbiome compositional differences between patients with primary hyperparathyroidism and osteoporosis, osteopenia, or normal BMD, gut microbiome composition was analyzed at phylum and species level (Fig. 6a, b). We also quantified overall community complexity (α diversity) and compositional similarity between samples (β diversity) at the species level. Patients with primary hyperparathyroidism and osteoporosis, osteopenia, or normal BMD had no significant differences in overall community complexity as measured by Shannon diversity (Fig. 6c) and microbial species beta diversity (Fig. 6d). As a hypothesis-generating tool, we next examined associations between variables. In the overall group of 50 primary hyperparathyroidism patients, spine BMD was significantly associated with 36 bacterial species and 1/3 radius BMD was associated with 35 bacterial species although no significant associations were found between species and spine or 1/3 radius BMD after FDR correction (Tables S3 and S4), Together, these data indicate that the presence of low BMD in patients with primary hyperparathyroidism is not associated with overall modifications of the composition of the gut microbiome, however, primary hyperparathyroidism may have caused more limited changes in the composition of the gut microbiome not identified by the analysis of alpha and beta diversity, which may be relevant for primary hyperparathyroidism-induced bone loss.

**Fig. 7:**
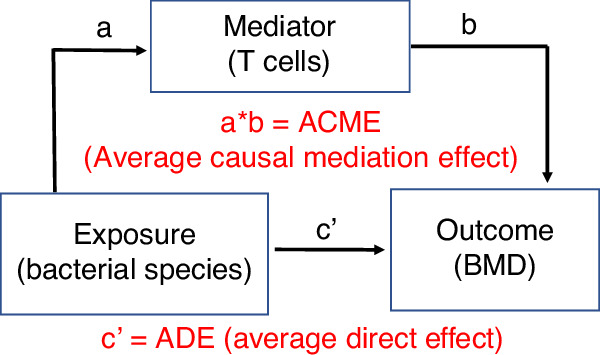
Schematic representation of a mediation analysis conducted to determine the existence of a cause-effect relationship between species (exposure) associated with *Tnf* and/or *Il17* transcript levels (mediators), and spine or 1/3 radius BMD (outcomes) in subjects with primary hyperparathyroidism

To investigate whether the microbiome influences BMD via an indirect mechanism, we analyzed the associations between bacterial species and T cell-produced TNF and IL-17. 29 species were associated with the relative frequency of TNF^+^ T cells, 38 species were associated with TNF^+^ T cells MFI, and 44 species with *Tnf* transcript levels. 26 species were associated with the frequency of Th17 cells, 54 species were associated with Th17 cell MFI, and 40 with *Il17* transcript levels. A mediation analysis was then performed^34^ to determine whether an exposure (bacterial species abundance) affected an outcome (BMD) through a mediator (TNF^+^ T cells, Th17 cells, TNF, or IL-17) (Fig. 7). After correction for age, sex, BMI, serum PTH, calcium and 25OHD levels, analysis of the species associated with TNF^+^ T cells, Th17 cells, TNF, or IL-17 revealed that *Bifidobacterium longum* was a significant mediator of the levels of PBMC *Il17* and *Tnf* transcripts (Table 3, and Table S5). *Il17 and/or Tnf* transcript levels were significant mediators of 1/3 radius BMD and 1/3 radius Ct.vBMD (Table 3, and Table S5). *Il17 and/or* *Tnf* transcript levels approached significance as mediators of spine BMD. Importantly, we detected significant average causal mediation effects (ACMEs) of *Bifidobacterium longum* on 1/3 radius BMD through *Il17* transcript levels and on Ct.vBMD through *Tnf* transcript levels (Table 3, and Table S5). *Bifidobacterium longum* had no average direct effects (ADEs) on 1/3 radius BMD. 1/3 radius Ct.vBMD or spinal BMD (Table 3, and Table S5). Correlations between the relative abundance of *Bifidobacterium longum* and the measured immune variables are shown in Fig. S6.

**Table 3:** *P* values of the mediation analysis shown in Fig. 5

Exposure	*Bifidobacterium longum*	
Mediator	PBMC *Il17* mRNA (relative expression)	PBMC *Tnf* mRNA (relative expression)
*P* value a	0.013*	0.001**
*P* value b 1/3 Radius BMD	0.034*	0.093
*P* value b 1/3 Radius Ct vBMD	0.415	0.041*
*P* value b Spine BMD	0.059	0.057
*P* value ACME 1/3 Radius BMD	0.034*	0.058
*P* value ACME 1/3 Radius Ct vBMD	0.432	0.032*
*P* value ACME Spine BMD	0.09	0.072
*P* value ADE 1/3 Radius BMD	0.51	0.634
*P* value ADE 1/3 Radius Ct vBMD	0.858	0.524
*P* value ADE Spine BMD	0.29	0.774

*Bifidobacterium longum was* a significant mediator of 1/3 radius BMD, 1/3 radius Ct vBMD, and spine BMD

*ACME* average causal mediation effect, *ADE* average direct effect

*P < 0.05 and **P < 0.01

To confirm the relationship between *Bifidobacterium longum* and BMD, we quantified *Bifidobacterium longum* abundance in stool samples from primary hyperparathyroidism patients with osteoporosis, osteopenia or normal bone density by PCR and found significant differences among these groups (Fig. S7a).

**Fig. 8:**
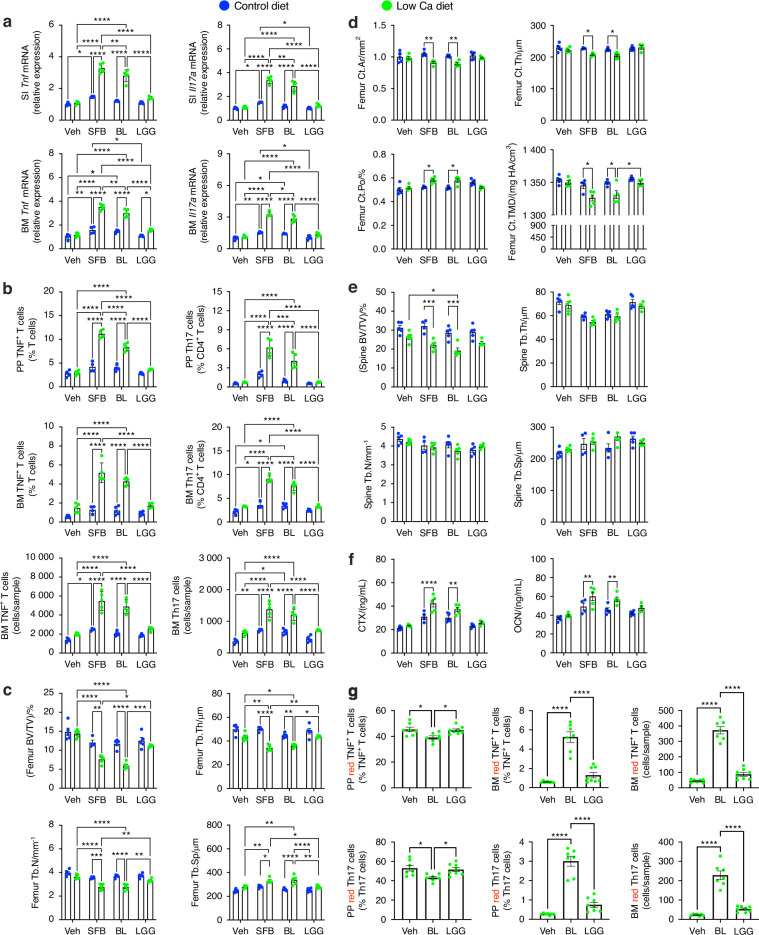
Monocolonization of germ-free mice with *Bifidobacterium longum* is sufficient for low calcium diet to increase *Tnf* and *Il17* transcript in the small intestine (SI) and BM, expand PP and BM TNF^+^ T cells and Th17 cells, induce cortical and trabecular bone loss, and increases the homing of PP TNF^+^ T cells and Th17 cells to the BM. WT germ-free mice monocolonized with SFB were positive controls. Germ-free mice monocolonized with vehicle or LGG were negative controls. **a** *Tnf* and *Il17* transcripts. **b** TNF^+^ T cells and Th17 cells. **c** Indices of femoral trabecular structure. The measured indices were trabecular bone volume fraction (BV/TV), trabecular thickness (Tb.Th), trabecular number (Tb.N), and trabecular separation (Tb.Sp). **d** Femoral indices of cortical structure and porosity. The measured indices were cortical area (Ct.Ar), cortical thickness (Ct.Th), cortical porosity (Ct.Po), and cortical tissue mineral density (Ct.TMD). **e** Spine indices of trabecular structure. **f** Serum level of CTX and OCN. **g** Frequency of PP and BM red fluorescent TNF^+^ T cells and Th17 cells from germ-free Kaede mice monocolonized with Vehicle, LGG, or *Bifidobacterium longum*. *n* = 4–7 mice/group. Data were expressed as mean ± SEM and were normally distributed according to the Shapiro–Wilk normality test. Data were analyzed by two-way ANOVA and post hoc tests applying the Bonferroni correction for multiple comparisons. *P < 0.05, **P < 0.01, ***P < 0.001, and ****P < 0.000 1. Non-significant comparisons not shown

To confirm that *Bifidobacterium longum* endows the capacity to expand TNF-producing T cells and to induce bone loss in response to PTH, we first quantified *Bifidobacterium longum* abundance in the stool microbiome of germ-free mice with stool microbiome originating from primary hyperparathyroidism patients with osteoporosis, osteopenia, or normal bone density by PCR and found significant differences among these groups (Fig. S7b). Subsequently, 4-week-old germ-free mice were monocolonized with either *Bifidobacterium longum*, SFB, or *Lactobacillus rhamnosus* GG (LGG). SFB represents a positive control as it is known to expand Th17 cells and promote PTH-mediated bone loss in mice,^14,24^ whereas LGG does not induce Th17 cells^27^ and was therefore used as a negative control. Mice were fed a standard sterile germ-free diet until 16 weeks of age, and then a low-calcium diet for 4 weeks to induce secondary hyperparathyroidism.^14^ Mice were housed in a Tecniplast ISOcageP Bioexclusion system^30^ to preserve the monocolonization status of these gnotobiotic mice. Measurements of *Bifidobacterium longum*-specific bacterial DNA by PCR revealed *Bifidobacterium longum* was present and had successfully colonized the germ-free mice (Fig. S8a). Attesting to the specificity of the monocolonization procedure, SFB bacterial DNA was undetectable in stool samples from germ-free mice monocolonized with *Bifidobacterium longum* (Fig. S8b). Low calcium diet increased the transcript levels of *Tnf* and *Il17* in the small intestine (SI) and BM of mice monocolonized with either *Bifidobacterium longum* or SFB, but not in those monocolonized with LGG (Fig. 8a). Low calcium diet also increased the frequency of PP and BM TNF^+^ T cells and Th17 cells in germ-free mice monocolonized with either *Bifidobacterium longum* or SFB, but not in those monocolonized with LGG (Fig. 8b). Further analysis of TNF^+^ T cells revealed that in mice monocolonized with either *Bifidobacterium longum* or SF, a low calcium diet increased the frequency of PP and BM CD4^+^TNF^+^ T cells, CD8^+^TNF^+^ T cells, and TNF^+^IL-17^+^ T cells (Fig. S9a–c). By contrast, mono-colonizing with *Bifidobacterium longum* or SFB did not increase the frequency of TNF^+^IFNγ^+^ T cells (Fig. S9d). Moreover, in germ-free mice monocolonized with *Bifidobacterium longum* or SFB but not LGG, low-calcium diet decreased femoral trabecular and cortical bone volume, area, and density, and altered trabecular and cortical bone structure and cortical porosity (Fig. 8c, d and Fig. S10a). Analysis of the spine by μCT revealed that low-calcium diet also decreased spinal BV/TV without altering trabecular structure in mice monocolonized with *Bifidobacterium longum* or SFB (Fig. 8e). Serum CTX, a marker of bone resorption, and serum OCN, marker of bone formation, were also increased by Low calcium diet in mice monocolonized with *Bifidobacterium longum* or SFB (Fig. 8f). Additional experiments were then conducted using germ-free Kaede mice monocolonized with *Bifidobacterium longum* or LGG. These studies revealed that *Bifidobacterium longum* but not LGG monocolonization decreased the relative frequency of PP KaedeR TNF^+^ T cells and Th17 cells and increased the frequency of BM KaedeR TNF^+^ T cells and Th17 cells (Fig. 8g), demonstrating that *Bifidobacterium longum* increases the trafficking of TNF^+^ T cells and Th17 cells from PPs to the BM.

**Fig. 9:**
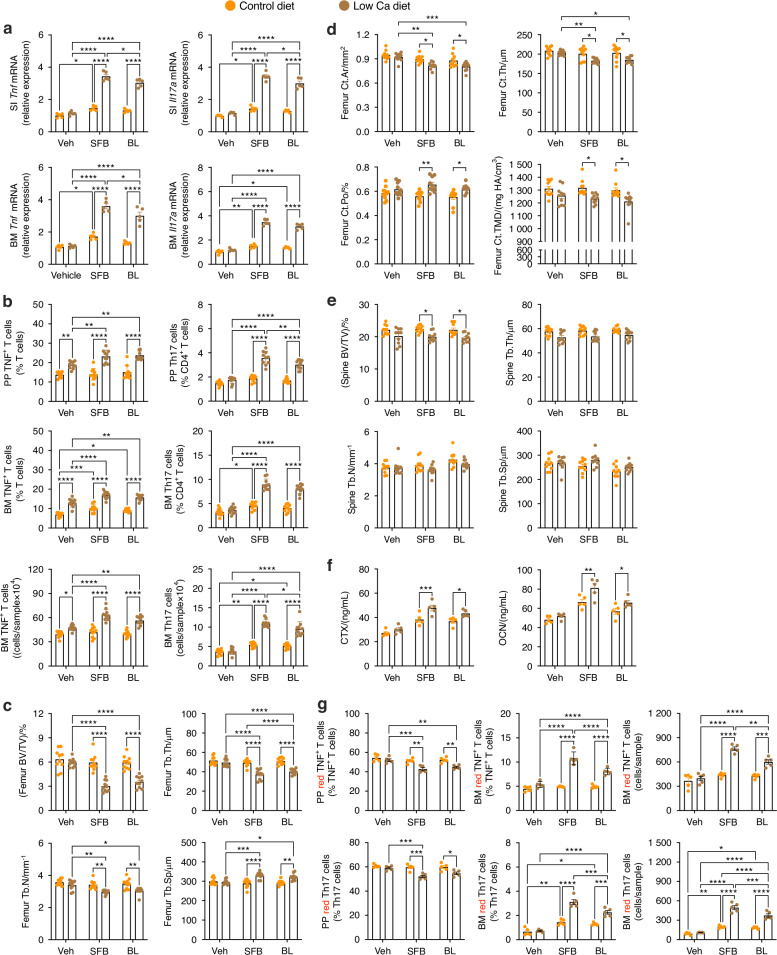
Daily oral supplementation of conventionally raised mice with *Bifidobacterium longum* potentiates the capacity of low calcium diet to increase *Tnf* and *Il17* transcript in the SI and BM, expand PP and BM TNF^+^ T cells and Th17 cells, and induce femoral bone loss, and increases the homing of PP TNF^+^ T cells and Th17 cells to the BM. Mice supplemented with SFB were positive controls. **a** *Tnf* and *Il17* transcripts. **b** TNF^+^ T cells and Th17 cells. **c** Femoral indices of trabecular structure: trabecular bone volume fraction (BV/TV), trabecular thickness (Tb.Th), trabecular number (Tb.N), and trabecular separation (Tb.Sp). **d** Femoral indices of cortical structure and porosity. Indices of cortical area: (Ct.Ar), cortical thickness (Ct.Th), cortical porosity (Ct.Po), and cortical tissue mineral density (Ct.TMD). **e** Spinal indices of trabecular structure. **f** Serum level of CTX and OCN. **g** Frequency of PP and BM red fluorescent TNF^+^ T cells and Th17 cells from Kaede mice. *n* = 5–10 mice/group. Data were expressed as mean ± SEM and were normally distributed according to the Shapiro–Wilk normality test. Data were analyzed by two-way ANOVA and post hoc tests applying the Bonferroni correction for multiple comparisons. *P < 0.05, **P < 0.01, ***P < 0.001, and ****P < 0.000 1. Non-significant comparisons not shown

To assess the role of *Bifidobacterium longum* in mice with a fully established microbiome, conventionally raised C57BL/6 SFB^−^ mice were gavaged daily with control *Bifidobacterium longum* or SFB from 16 to 20 weeks of age and fed a low calcium diet or a normal calcium diet. Analysis of bacterial DNA by PCR revealed that daily gavaging of *Bifidobacterium longum* was successful in increasing its abundance in the stool of mice (Fig. S8c). A low-calcium diet increased *Tnf* and *Il17* transcript levels in the SI and BM, and the frequency of PP and BM TNF^+^ T cells and Th17 cells in mice supplemented with either *Bifidobacterium longum* or SFB, but not in control mice gavaged with vehicle (Fig. 9a, b). In mice gavaged with either *Bifidobacterium longum* or SFB Low calcium diet increased the frequency of PP and BM CD4^+^TNF^+^ T cells, CD8^+^TNF^+^ T cells, and TNF^+^IL-17^+^ T cells (Fig. S11a–c). By contrast, gavaging *Bifidobacterium longum* or SFB did not increase the frequency of TNF^+^IFNγ^+^ T cells (Fig. S11d). Analysis of the femur by μCT revealed that low-calcium diet decreased trabecular and cortical bone volume, altered trabecular structure, and cortical area, porosity and tissue mineral density in mice gavaged with or SFB, (Fig. 9c, d and Fig. S10b). Moreover, in mice gavaged with *Bifidobacterium longum* or SFB low-calcium diet decreased spinal BV/TV without significant changes in spinal trabecular structure (Fig. 9e) and increased the serum level of CTX and OCN (Fig. 9f). Moreover, a low-calcium diet decreased the relative frequency of PP KaedeR TNF^+^ T cells and Th17 cells and increased the frequency of BM KaedeR TNF^+^ T cells and Th17 cells in mice supplemented with either *Bifidobacterium longum* or SFB mice, but not in those gavaged with vehicle (Fig. 9g). These findings confirmed that the presence of *Bifidobacterium longum* in the gut microbiome allows PTH to cause the expansion and migration of TNF^+^ T cells and Th17 cells and to induce bone loss.

## Discussion

We report that the bone-wasting effect of primary hyperparathyroidism was transmissible via the gut microbiome. Mediation analysis identified *Bifidobacterium longum* as the primary contributing species. Animal studies confirmed the role of *Bifidobacterium longum* in the bone loss induced by hyperparathyroidism.

*Bifidobacterium longum* promoted intestinal TNF^+^ T cell activation and migration to the BM, events which in mice are followed by TNF-driven, chemokine-mediated recruitment of intestinal Th17 cells to the BM.^14^ Accordingly, the frequency of circulating TNF^+^ T cells and Th17 cells, along with their TNF and IL-17 expression and MFI, predicted BMD and bone structure in subjects with primary hyperparathyroidism. Thus, both the expansion of these lineages and their increased production of TNF and IL-17 contributed to the bone loss induced by primary hyperparathyroidism.

TNF^+^ T cells and TNF production were strong predictors of BMD, and cortical and trabecular bone volume at all sites, whereas Th17 cells and IL-17 production correlated with BMD at most sites and with radius cortical bone. These findings strongly implicate T cell-produced TNF and IL-17 in primary hyperparathyroidism-induced bone loss. However, further studies will be necessary to conclusively establish the causal role of these cytokines in humans.

In mice, T cells are the source of a pool of TNF and IL-17 critical for PTH-induced bone loss, as PTH does not result in bone loss in mice specifically lacking T cell production of these cytokines.^9,12^ IL-17 potently promotes osteoclastogenesis by stimulating the production of inflammatory cytokines by macrophages and other cells,^35,36^ and the expression of RANKL by osteocytes.^13,37^ Attesting to the mechanistic relevance of IL-17 induced RANKL expression by murine osteocytes, deletion of the IL-17 receptor in osteocytes prevents PTH-induced bone loss.^13^

Human and animal studies show that the gut microbiome regulates the response to sex-hormone deficiency,^28,33,38^ BMD,^38^ bone tissue material properties,^39^ skeletal development,^40^ and pathogenesis of osteoporosis.^41^ As the microbiome is also crucial for PTH-induced bone loss via TNF^+^ T cells and Th17 cell expansion in mice,^14^ we studied its relevance by transferring stool from primary hyperparathyroidism subjects to wild-type and Kaede germ-free mice with secondary hyperparathyroidism induced by a low-calcium diet. This strategy induces bone loss similarly to cPTH infusion,^14,29^ which is technically challenging to model in germ-free mice. Our findings revealed that the microbiome mediated the skeletal effects of primary hyperparathyroidism and regulated TNF^+^ T cell and Th17 cell expansion and migration from the gut to BM.

It should be noted that while we did not detect differences in microbiome alpha and beta diversity between patients with osteoporosis, osteopenia, or normal BMD, differences in alpha and beta diversity were found in mice with microbiome originating from these three groups. We hypothesize that subtle compositional and functional differences between the three groups of donors, such as strain-level variation or differential abundance of low-frequency taxa, may have influenced colonization efficiency in the murine gut. These differences may have been amplified by the strong ecological filtering imposed by the mouse host environment, which favors certain taxa based on host physiology, leading to significant differences in alpha and beta diversity between the three groups of recipient mice.^42,43^

The gut microbiome has been reported to regulate intestinal calcium absorption both directly and via vitamin D.^44^ In this study, we did not measure calcium absorption, serum calcium, phosphate, PTH, and 25OHD levels in recipient mice. Therefore, we cannot exclude the possibility that the gut microbiome may condition the response to PTH not only through immune modulation but also by altering calcium absorption.

Patients with primary hyperparathyroidism and either normal BMD, osteopenia, or osteoporosis had similar overall microbiome composition, indicating that the propensity of primary hyperparathyroidism to induce bone loss may depend on the relative abundance of a single or a few species capable of expanding TNF^+^ T cells and/or Th17 cells. Several pathogens and over 20 non-virulent gut bacterial strains have been identified that induce Th17 cell differentiation in the human gut.^26,27^ An even larger number of pathogens and saprophytes induce TNF^+^ T cells, including *Escherichia coli*,^19^ *Salmonella enterica*,^20^ *Helicobacter pylori*,^21^ and *Lactobacillus*.^22^ Accordingly, we found significant associations between many microbial species and TNF^+^ T cells or Th17 cells.

In mice, monocolonization of germ-free hosts with *Bifidobacterium longum* or supplementation of conventional mice with *Bifidobacterium longum* expanded intestinal TNF⁺ T cells and Th17 cells, promoted their migration to the BM, and induced PTH-dependent bone loss. Importantly, *Bifidobacterium longum*-driven bone loss occurred only under low calcium conditions, indicating that *Bifidobacterium longum* promotes bone loss only in concert with elevated PTH. It is also likely that only some strains of *Bifidobacterium longum*, such as the strain used in this study (*Bifidobacterium longum subsp. Longum*, ATCC 15707), activate T cells and drive bone loss, whereas others may not.

*Bifidobacterium longum* has been used as a probiotic in a variety of conditions. For example, it was found to improve sleep quality^45^ and to protect against inflammatory bowel disease.^46^ In mice, *Bifidobacterium longum* attenuated ovariectomy-induced bone loss^47^ and to improved age-related delays in fracture repair.^48^ However, the effects of *Bifidobacterium longum* supplementation in PTH-induced bone loss have not been previously reported. We found that in primary hyperparathyroidism patients, the frequency of *Bifidobacterium longum* predicted low bone density. In mice, supplementation with a specific strain of *Bifidobacterium longum* promoted PTH-induced bone loss. The differential effects of *Bifidobacterium longum* in various disease states might be related to the strain of *Bifidobacterium longum* used as probiotic and to its capacity to induce the differentiation of either bone-wasting cells, such as TNF^+^ T cells, or bone-sparing T cells, such as regulatory T cells. In line with our observation, previous researchers have found strain-specific immunomodulatory effects of *Bifidobacterium* species exhibiting distinct interactions with host immunity and immune responses.^49–51^ The mechanism by which *Bifidobacterium longum* promotes the expansion of intestinal TNF-producing T cell remains unknown. Based on the mechanism of action of other bacteria, we speculate that direct activation of intestinal immune cells by a bacterial product is the likely mechanism. However, it is also possible that specific strains of *Bifidobacterium longum* release metabolites sensed by intestinal dendritic cells, naïve T cells, or other immune cells involved in the differentiation and expansion of TNF^+^ T cells and Th17 cells in gut tissue.^52,53^

DXA provides integrated measurements of trabecular and cortical bone. However, the lack of bone compartment-specific analysis with DXA limits its sensitivity in detecting bone loss in clinical disorders that predominantly affect cortical bone, such as primary hyperparathyroidism, or trabecular bone, such as the early post-menopausal period. By contrast, HRpQCT generates specific indices of trabecular and cortical volume, density, and structure at a given skeletal site. Using HRpQCT, we were able to successfully correlate TNF^+^ T cell and Th17 cell frequency and cytokine production with indices of trabecular and cortical bone volume and structure.

Study limitations include the use of stool bacteria that may not fully represent the small intestine’s microbiome, as some taxa are mucus-associated and may not be abundant in stool. Another limitation is that our participants were drawn from a single site and reflect a narrow demographic comprising mainly Caucasian women. Because microbiota is dependent in part on demographic factors, this study may not be representative of other populations. Future studies should include other racial and ethnic groups and should determine if *Bifidobacterium longum* is a universal driver of primary hyperparathyroidism-induced bone loss, or if other microbial taxa drive bone loss in specific populations. Future studies are also required to ascertain if natural variation in microbiome composition due to diet, genetics, or geographic location confers protection against primary hyperparathyroidism-induced bone loss in specific groups.

Our approach to mono-colonizing germ-free mice with *Bifidobacterium longum* involved housing mice in hermetically sealed, positive-pressure ISO cages within the Emory Gnotobiotic Animal Core (EGAC), which adheres to strict standard operating procedures to prevent environmental contamination and ensure monocolonization. We confirmed successful colonization with *Bifidobacterium longum* using species-specific 16S qPCR. However, we acknowledge a limitation of this verification method in that additional microbes could theoretically colonize the mice without being detected by *Bifidobacterium longum*-specific primers. Although this scenario is unlikely given the controlled conditions, future studies should incorporate comprehensive 16S rRNA sequencing of fecal samples from monocolonized mice to fully validate the absence of other taxa.

In conclusion, our findings provide evidence that, as suggested by our pre-clinical studies, increased production of TNF and IL-17 by T cells is a pivotal mechanism of bone loss in primary hyperparathyroidism subjects. *Bifidobacterium longum*, a taxon frequently found in the gut microbiome, drives PTH-dependent TNF^+^ T cell differentiation and migration to the BM, events required for microbiome-induced intestinal Th17 cell homing and functional activation in the BM, thus contributing to the bone-wasting effects of primary hyperparathyroidism. Our findings suggest that microbiome composition helps to explain the heterogeneous skeletal phenotype of subjects with primary hyperparathyroidism; thus, microbiome sequencing may help identify those at risk for bone loss and fractures. Modifying the microbiome with antibiotics or precision probiotics may offer a potential preventive and therapeutic approach to mitigate the damaging skeletal effects of primary hyperparathyroidism.

## Materials and methods

### Sex as a biological variable

Our study examined male and female subjects and female mice.

### Subjects and clinical data

Men and women aged 30–85 were eligible for inclusion if they had a confirmed diagnosis of primary hyperparathyroidism at screening. Screening was conducted at Columbia University, NY. The diagnosis of primary hyperparathyroidism was established based on elevated circulating levels of albumin-corrected total serum calcium or elevated ionized calcium with PTH levels that were frankly elevated or inappropriately normal in the presence of hypercalcemia in at least two instances. Inclusion criteria were as follows: confirmed diagnosis of primary hyperparathyroidism, presence of normal renal function (estimated glomerular filtration rate [eGFR] > 60 cc/min), BMI ≥ 18 and ≤33 kg/m^2^, willing and able to give written informed consent study participation, commitment not to use any products that may influence the study outcome, ability to understand and comply with the requirements of the study. Exclusion criteria were the presence of at least one of the following conditions: conditions that prevent the execution or alter the results of DXA or HRpQCT measurements, type 1 and type 2 diabetes mellitus, history of bariatric surgery or other forms of malabsorption, excessive alcohol use (defined as more than three alcoholic drinks per day), eGFR ≤ 60 cc/min, clinically significant cardiovascular disease, clinically significant liver disease, any malignancies, other than localized skin squamous/basal cell carcinoma, diagnosed within the previous 5 years, any history of metastatic cancer, history of use of oral supplement products containing probiotic bacteria (more than once per week) within 4 weeks prior to study visit, current use (within the past 8 weeks) of any medication known to influence the immune or skeletal system, current use or use in the last 5 years of oral or injectable bisphosphonates, current or use within 1 year of other drugs to prevent or treat osteoporosis, use of antibiotics during the previous 2 months or frequent use of antibiotics (>2 courses during the previous 12 months) for any reason, use of nicotine-containing products during the last 6 months, chronic inflammatory conditions such as rheumatoid arthritis, psoriasis, or inflammatory bowel disease, secondary hyperparathyroidism, use of glucocorticoids or cinacalcet, or any other condition known to affect Th17 cells, IL-17 production, PTH production, or calcium and phosphate levels, 25(OH)D < 12 ng/mL at time of screening, history of hypogonadism. Demographic characteristics, obtained from the medical record, were confirmed by the study subject. Medical history, family history, fracture history, tobacco, and alcohol history were obtained at the study visit via IRB-approved questionnaires.

Fasting morning labs were drawn before 11 AM and analyzed by Quest Diagnostics for serum electrolytes, renal, and hepatic function. Serum 25OHD was measured by CLIA (Diasorin, Stillwater, MN; nl ≥ 20 ng/mL). Serum PTH 1-84 was measured by ELISA (Quidel, Athens, OH). Serum CTX was measured by ELISA (Euroimmun, Mountain Lakes, NJ). Serum P1NP was measured by RIA using (Euroimmun, Mountain Lakes, NJ).

### Stool collection

Eligible participants were provided with stool collection kits (Fisherbrand Commode Specimen Collection System) and instructed to collect a stool sample within 48 h before their scheduled visit. Participants, instructed to keep their stool samples frozen at −20 °C, were given ice packs and a cooler for sample transportation. Once received, stool samples were stored at −80 °C before being shipped overnight on dry ice to Emory University. All samples were received in the frozen state.

### DXA and HRpQCT

BMD of the lumbar spine (L1-L4), total hip, FN, and 1/3 radius were measured by DXA at Columbia University (Horizon; Hologic Inc., Waltham, MA) or locally (Lunar or Hologic), if DXA was obtained within 3 months of the study visit. Participants were scanned at all skeletal sites unless contraindications, such as the presence of hardware, precluded analysis. In vivo precision was 1.1% at the spine, 1.24% at total hip, 2.4% at femoral neck, and 1.8% at the forearm. Using the manufacturer’s reference norms, BMD was expressed in g/cm^2^ and T scores.

HRpQCT was performed using a Scanco Medical (Brüttisellen, Switzerland) second-generation scanner (XCT2) with a nominal isotropic voxel size of 61 μm (68 kVp voltage, 1 460 μA current, 43 s integration time). Scans were obtained at the non-dominant limb; opposite side was scanned if there was a contraindication. The region of interest (ROI) was identified according to established guidelines.^54^ Briefly, a reference line was defined on a 2D scout image, and distal radius and tibia scans were acquired at a fixed offset of 9 mm and 22 mm from the reference line, resulting in a 10.2 mm axially long scan region. A more proximal diaphyseal radius scan was also obtained using the relative offset protocol (30% of ulnar length) in order to obtain a more cortical rich scan region.^54^ Ulnar length was used as a proxy for radial length, as ulnar ends are easier to identify. A single trained operator acquired and analyzed all the scans, and scans with a motion score >3 were excluded from analysis.^55^ For quality control, a standardized phantom provided by the manufacturer was scanned daily. In vivo short-term reproducibility was between 0–5% for all measures except for Ct.Po, which was 9.9% and 15.6% at the distal tibia and radius, respectively.

### Mice

Conventional C57BL/6J SFB^−^ mice were purchased from the Jackson Laboratory (Bar Harbor, ME). Kaede mice were purchased from RIKEN Bioresource Research Center. Kaede mice were re-derived to germ-free status by Taconic Biosciences (Rensselaer, NY). Germ-free C57BL/6NTac mice were purchased from Taconic Biosciences. Conventional mice were housed at Emory University. All germ-free mice were maintained by the Emory Gnotobiotic Animal facility.

All mice were acclimatized within our facility for 3 days before experimentation. Littermates of the same sex were randomly assigned to experimental groups.

### Mouse diet

Mice were fed sterilized 5V5R chow containing 0.99% calcium (LabDiet, St. Louis, MO). Mice on a Low calcium diet were fed sterilized food made by MP Biomedicals (Solon, OH) containing either 0.01% calcium (low calcium diet) or 1.75% calcium (control diet) for 4 weeks. All mice received autoclaved water ad libitum.

### Bacterial supplementation and generation of monocolonized mice

To generate conventional SFB^+^ mice and to mono-colonize germ-free mice with SFB, liquid suspensions of fecal pellets from SFB mono-associated mice were orally gavaged to SFB^−^ conventional or germ-free mice as described.^33,56^ SFB status was verified by qPCR using specific SFB 16S rDNA gene primers 5′-GACGCTGAGGCATGAGAGCAT-3′, forward; and 5′-GACGGCACGGATTGTTATTCA-3′, reverse; and total bacterial 16S rRNA, 5′-GTGCCAGCMGCCGCGGTAA-3′, forward, and 5′-GGACTACHVGGGTWTCTAAT-3′, reverse.^56^ For enrichment of *Bifidobacterium longum* *(Bifidobacterium longum subsp. Longum*, ATCC 15707) or LGG (ATCC 53103), 16-week-old conventional SFB^−^ mice were orally gavaged with 100 μl of a liquid preparation containing *Bifidobacterium longum* or LGG at 1 × 10^9^ CFU/100 µL water, 5 days per week for 4 weeks. Germ-free mice were monocolonized with *Bifidobacterium longum* or LGG at 4 weeks of age by oral gavage with *Bifidobacterium longum* or LGG (1 × 10^9^ CFU/100 µL water) three times over 6 days. The *Bifidobacterium longum* status was confirmed by quantitative qPCR using *Bifidobacterium longum*-specific 16S rDNA gene primers: forward primer 5′-CTCCTGGAAACGGGTGG-3′ and reverse primer 5′-GGTGTTCTTCCCGATATCTACA-3′. Monocolonized mice were housed in a Tecniplast ISOcage P-Bioexclusion System.^30^

### Human microbiome-associated mice generation

Human microbiome-associated mice were generated as described.^57–59^ Briefly, 4-week-old germ-free C57BL/6 or germ-free Kaede mice were gavaged three times over 6 days with 100 µL of fecal suspensions collected from primary hyperparathyroidism patients.

### Cell trafficking studies

SFB^−^ Kaede mice [B6.Cg-c/c Tg(CAG-tdKaede)15Utr] were purchased from RIKEN Bioresource Research Center and sent to Taconic Biosciences for germ-free IVF rederivation to develop germ-free Kaede mice. Pregnant germ-free females were received at Emory Gnotobiotic Animal Core, and their offspring were confirmed to be germ-free before being used for breeding to expand the colony of germ-free Kaede mice. To generate SFB^+^ Kaede mice, conventional SFB^−^ Kaede mice were gavaged with fecal suspension from SFB mono-associated mice.^28,33,56^ Kaede mice express a photoconvertible fluorescence protein that shifts from green (518 nm) to red (582 nm) upon exposure to near-UV (350–410 nm) light, as previously described.^28,33,56^ Kaede mice underwent laparotomy as described,^28,33,56^ whereupon 4 PPs were photoconverted by exposure to near-UV light (390 nm) for 2 min each, and housed in Tecniplast ISOcageP Bioexclusion cages.^30^ Mice were sacrificed 24 h after photoconversion, and PP and BM cells were collected. Red fluorescing TNF^+^ T cells and Th17 cells were enumerated by flow cytometry.

### Mouse μCT measurements

μCT scanning and analysis of the distal femur and spine were performed as reported previously,^28,33,60^ using a Scanco μCT 50 scanner (Scanco Medical). All the bones were evaluated using 12 μm isotropic voxels. For L4 vertebrae (spine), 100 tomographic slices of trabecular bone were analyzed. For the femoral trabecular region, we analyzed 70 slices from the starting 20 slices below the distal growth plate. Femoral cortical bone was assessed using 80 continuous CT slices located at the femoral midshaft. The X-ray tube potential was 70 kVp, and the integration time was 200 ms. Thresholding was done according to Bouxsein et al.^61^ as recommended by Scanco Medical. The same threshold value was used for all measurements.

### Human TNF^+^ T cells and Th17 cells quantification by flow cytometry

The number of circulating TNF^+^ T cells and Th17 cells, and their MFI, were determined by flow cytometry. See the gating strategy in (Fig. S1). PBMCs were isolated using All Cell Preparation Tubes. For surface staining of PBMCs or murine cells, cells were stained with, Human TruStain FcX™ (Fc Receptor Blocking Solution) (catalog 422302), anti-human APC-CD3 (clone OKT3, catalog 317318), FITC-CD4 (clone RPA-T4, catalog 300506), anti-APC-Mouse IgG2a Ab, κ Isotype Ctrl antibody for CD3 (clone MOPC-173, catalog 400219), anti-FITC-Mouse IgG1 Ab, κ Isotype Ctrl antibody for CD4 (clone MOPC-21, catalog 400109), The live cells were discriminated using the Zombie NIR Fixable Viability Kit (BioLegend). Anti-FITC-Mouse IgG1 Ab, κ Isotype Ctrl antibody for IL-17a and TNF-α (clone MOPC-21, catalog 400157) (all from BioLegend). For intracellular staining, cells were incubated with an activation cocktail containing Monensin (Biolegend), followed by intracellular fixation & permeabilization using a Thermo Fisher Scientific kit. Anti-human BV421-IL-17A (clone BL168, catalog 512322) and BV421-TNF-α (clone MAb11, catalog 502932) (all from Biolegend). Flow cytometry was performed on a BD LSR2 (BD Biosciences), with analysis using FlowJo (Tree Star Inc.).

### Preparation of PP and BM single-cell suspension

PP cell isolation was performed as described.^28,56^ Briefly, the SI was removed and flushed of fecal content. PPs were excised and collected in 1 mL cooled RPMI1640 (Corning). PPs were dissociated using a 2.5 mL syringe plunger and gently forced through a 70 µm cell strainer placed over a 50 mL tube (Corning). A single-cell suspension was used for analysis by flow cytometry. The femur, tibia, and pelvic bones were flushed with PBS for BM cell isolation, and BM cells were collected. Red cells were removed with red blood cell lysis buffer (Biolegend) before antibody staining. Single-cell suspensions of BM cells were used for analysis by flow cytometry, as previously described.^14,28,56^

### Flow cytometry of murine cells

For cell surface staining, cells were stained with anti-mouse purified CD16/32 (Fc blocking Ab, clone 93, catalog 101302), BV 510-CD45 (clone 30-F11, catalog 103138), BV 421-TCRβ (clone H57-597, catalog 109230), AF 700-CD3 (clone 17A2, catalog 100216), PerCP/Cy5.5-CD4 (clone RM4-5, catalog 100540), BV 711-CD8 (clone 53-6.7, catalog 100748) (Biolegend) and FITC-Vβ T cell receptor (clone RR4-7, catalog 553193) (BD Biosciences). The live cells were discriminated by Zombie NIR Fixable Viability Kit (BioLegend) or LIVE/DEAD Fixable Yellow Dead Cell Stain Kit (Thermo Fisher Scientific). For intracellular staining, cells were incubated with cell activation cocktail (Biolegend) in the presence of Monensin Solution at 37 °C for 12 h. Anti-mouse PE or APC-IL-17A (clone eBio17B7, catalog 17-7177-81 or 12-177-81) (Thermo Fisher Scientific), APC or BV650- TNF (clone MP6-XT22, catalog 506308 or 506333), PE/Cyanine7-IFN-γ (clone XMG1.2, catalog 505826) (Biolegend) were added after cell fixation and permeabilization with Intracellular Fixation & Permeabilization Buffer Set (Thermo Fisher Scientific). Flow cytometry was performed on a BD FACSymphony A5 system (BD Biosciences), and data were analyzed using FlowJo software (Tree Star Inc.).

### Real-time PCR and murine primers

Total RNA was isolated from murine BM and SI using TRIzol reagent (Thermo Fisher Scientific) and DNase Max kit (QIAGEN) according to the manufacturer’s directions. RNA was reverse transcribed to cDNA using a High-Capacity Reverse Transcription kit and quantified by qPCR using the StepOnePlus Real-Time PCR system. The primers used were *18S* rRNA, 5′-ATTCGAACGTCTGCCCTATCA-3′ (forward) and 5′-GTCACCCGTGGTCACCATG-3′ (reverse); *Tnf*, 5′-AACTCCAGGCGGTGCCT AT-3′ (forward) and 5′-TGCCACAAGCAGGAATGAGA-3′ (reverse); *Il17a*, 5′-TGACGCCCACCTACAACATC-3′ (forward) and 5′-CATCATGCAGTTCCGTCA GC-3′ (reverse). All reagents/kits were from Applied Biosystems. Relative expression was calculated using the 2^−ΔΔCT^ method with normalization to 18S.

### Human *Il17a* and *Tnf* transcript measurements

Total RNA from PBMCs was isolated using TRIzol reagent (Thermo Fisher Scientific) and the DNase Max kit (QIAGEN) from PBMC. RNA was reverse transcribed to cDNA using a High-Capacity Reverse Transcription kit and quantified by qPCR using the StepOnePlus Real-Time PCR system. The primers used for human PBMCs were *Il17a*, 5′-TCCCACGAAATCCAGGATGC-3′ (forward) and 5′-GGATGTTCAGGTTGACCATCAC-3′ (reverse); for human *Tnf*, 5′TGCTCCTCACCCACACCAT-3′ (forward) and 5′-GGAGGTTGACCTTGGTCTGGTA-3′ (reverse); for human *β-actin*, 5′-GTTGCTATCCAGGCTGTG-3′ (forward) and 5′-TGATCTTGATCTTCATTGTG-3′ (reverse). All reagents/kits were from Applied Biosystems. Relative expression was calculated using the 2^−ΔΔCT^ method with normalization to *β-actin*.

### Metagenomic shotgun microbiome sequencing and bioinformatics analysis

Microbiome composition was determined by a metagenomic shotgun sequencing methodology developed by CosmosID Inc. Briefly, DNA from fecal samples was isolated using the QIAGEN DNeasy PowerSoil Pro Kit. DNA libraries were prepared using the Illumina Nextera XT library preparation kit, and the library quantity was assessed with Qubit (Thermo Fisher). Libraries were sequenced on an Illumina HiSeq platform 2 × 150 bp. The average sequencing depth was 4 948 936 reads per sample (range: 1 240 758–14 788 148). Taxonomic assignment and functional analysis were performed using the CosmosID bioinformatics platform. Taxonomy was assigned using the kmer-based algorithms and the GenBook® database, which comprises >150 000 bacteria, viruses, fungi, and protists genomes. The principal software pipeline has been optimized for processing unmapped and unaligned raw sequence reads of lengths less than 100 bp.

### Statistical analysis

Normally distributed data according to the Shapiro–Wilk normality test were analyzed by Pearson correlation, simple linear regression, unpaired two-tailed *t*-tests, one-way ANOVA, or two-way ANOVA as appropriate, with Bonferroni correction for multiple comparisons. Kruskal–Wallis test was used to test significant differences in alpha diversity. PERMANOVA multivariate analysis was used to test significant differences between groups in the beta diversity analysis. Data that were not normally distributed were analyzed using Spearman correlations. The Linear Decomposition Model (LDM) was implemented using the R package LDM (v.6.0.1) to test the association between the relative abundance transformed composition of the microbiome at the species level and explanatory variables. All LDM models were adjusted for age and sex. Significant species were reported with an adjusted *P* value < 0.05. The relative abundance of *Bifidobacterium longum* was tested for mediation of its effect on bone density and volume through TNF^+^ T cells, Th17 cells, and *Tnf/Il17* mRNA levels, using causal mediation analysis.^34^ The relative abundance of all significant species determined by LDM modeling with a *P* value < 0.05 was also tested in mediation analysis.^34^ The analysis was performed using the mediate function from the mediation R package (v.4.5.0) with 1 000 simulations to estimate the Average Causal Mediation Effect (ACME) and Average Direct Effect (ADE).^34^ We applied an isometric log-ratio (ILR) transformation to the relative abundance of each species, adjusted the analysis for BMI, PTH, serum calcium, and serum 25OHD levels, and incorporated medsens from the mediation R package for each mediation analysis. We also incorporated 10 000 bootstrap simulations in each mediation analysis to provide more stable ACME and ADE confidence intervals. The estimated coefficients (betas) were reported as an indicator of the effect size. *P* and *q* values < 0.05 were considered significant.

### Study approval

Human studies were approved by the IRB of Columbia University and Emory University. All subjects provided written informed consent. All animal procedures were approved by the Institutional Animal Care and Use Committee of Emory University in compliance with all applicable federal regulations governing the protection of animals in research.

## Supplementary information

Supplementary Material

## Data Availability

All data sets, and all R and Python codes used in the analysis are available at: https://github.com/SanchitiPatil16/PHPT.githyperparathyroidism.git. Microbiome sequencing reads are deposited at SRA: SubmissionID: SUB14888249. BioProject ID: PRJNA1193041. Data are also available from the corresponding author upon reasonable request.

## Software

Flow cytometry data were analyzed using FlowJo v.10.9.0 (Tree Star) software. Graphics and statistics were created with GraphPad Prism software v.10.4.0.
