# Supplementary material for: Bacterial specificity of the gut microbiome predicts bone density in primary hyperparathyroidism
Source: Bone Res. 2026 May 25;14:57. doi: 10.1038/s41413-026-00529-1 (PMC13201753; doi:10.1038/s41413-026-00529-1)
Supplement: Supplementary file 1 — Supplementary Material [file 41413_2026_529_MOESM1_ESM.docx]

**Supplementary Table 1. r values and p values of the correlations between BMD, HRpQCT indices, TNF^+^ T-cells (frequency and MFI), Th17 cells (frequency and MFI), and PBMC *Tnf* and *Il17* transcripts levels in the entire study population.**

|  | **TNF^+^ T cells (%)** | | **TNF^+^ T cells (MFI)** | | **PBMC Tnf mRNA** | | **Th17 cells (%)** | | **Th17 cells (MFI)** | | **PBMC Il17 mRNA** | |
| --- | --- | --- | --- | --- | --- | --- | --- | --- | --- | --- | --- | --- |
|  | p value | r value | p value | r value | p value | r value | p value | r value | p value | r value | p value | r value |
| **Spine BMD** | ****0.003** | -0.417 | ****0.006** | -0.391 | ****0.001** | -0.443 | ***0.041** | -0.293 | ***0.046** | -0.286 | ****0.007** | -0.286 |
| **1/3 Radius BMD** | ****0.009** | -0.368 | ***0.019** | -0.335 | ****0.002** | -0.425 | ***0.022** | -0.326 | 0.087 | -0.247 | ****0.004** | -0.247 |
| **Total Hip BMD** | 0.100 | -0.240 | 0.153 | -0.210 | ****0.008** | -0.376 | 0.115 | -0.230 | ***0.015** | -0.361 | ***0.016** | -0.361 |
| **FN BMD** | ***0.016** | -0.346 | ***0.023** | -0.327 | ****0.007** | -0.379 | 0.054 | -0.280 | 0.119 | -0.228 | ***0.047** | -0.228 |
|  |  |  |  |  |  |  |  |  |  |  |  |  |
| **1/3 Radius Tt.vBMD** | ***0.023** | -0.324 | ****0.010** | -0.366 | ****0.001** | -0.446 | ****0.005** | -0.393 | ****0.008** | -0.374 | ***0.017** | -0.374 |
| **1/3 Radius Tb.vBMD** | 0.934 | 0.012 | 0.053 | -0.278 | 0.507 | 0.096 | 0.893 | 0.020 | 0.526 | 0.093 | 0.560 | 0.093 |
| **1/3 Radius BV/TV** | 0.282 | -0.157 | 0.560 | -0.085 | 0.912 | 0.016 | 0.662 | 0.064 | ***0.015** | 0.346 | 0.823 | 0.346 |
| **1/3 Radius Tb.N** | ***0.046** | -0.286 | ***0.017** | -0.340 | 0.212 | -0.180 | 0.242 | -0.170 | 0.364 | -0.133 | 0.119 | -0.133 |
| **1/3 Radius Tb.Th** | 0.364 | 0.132 | 0.701 | -0.056 | ***0.050** | 0.279 | 0.262 | 0.163 | ***0.011** | 0.358 | 0.353 | 0.358 |
| **1/3 Radius Tb.Sp** | 0.054 | 0.277 | ***0.012** | 0.355 | 0.138 | 0.213 | 0.149 | 0.209 | 0.116 | 0.227 | ***0.028** | 0.227 |
| **1/3 Radius Ct.vBMD** | 0.271 | -0.160 | 0.406 | -0.121 | ****0.003** | -0.413 | 0.053 | -0.279 | 0.277 | -0.159 | 0.102 | -0.159 |
| **1/3 Radius Ct.Th** | ***0.017** | -0.339 | ***0.036** | -0.300 | ****0.003** | -0.414 | ****0.008** | -0.376 | ***0.023** | -0.324 | ****0.005** | -0.324 |
| **1/3 Radius Ct.Po** | ***0.019** | 0.333 | 0.350 | 0.136 | *****0.001** | 0.473 | ***0.011** | 0.360 | ****0.001** | 0.445 | 0.155 | 0.445 |
|  |  |  |  |  |  |  |  |  |  |  |  |  |
| **UD Radius Tt.vBMD** | ****0.002** | -0.435 | *****<0.001** | -0.502 | ****0.001** | -0.451 | 0.056 | -0.275 | 0.089 | -0.245 | 0.153 | -0.245 |
| **UD Radius Tb.vBMD** | ****0.003** | -0.412 | ****0.007** | -0.379 | ******<0.001** | -0.501 | 0.088 | -0.246 | 0.407 | -0.121 | ***0.011** | -0.121 |
| **UD Radius BV/TV** | ****0.009** | -0.370 | ***0.020** | -0.331 | *****0.001** | -0.473 | 0.137 | -0.215 | 0.610 | -0.075 | ***0.014** | -0.075 |
| **UD Radius Tb.N** | ****0.001** | -0.448 | ****0.004** | -0.402 | ******<0.001** | -0.491 | ***0.031** | -0.308 | 0.215 | -0.180 | ***0.020** | -0.180 |
| **UD Radius Tb.Th** | 0.769 | -0.043 | 0.818 | -0.034 | 0.596 | -0.077 | 0.347 | 0.137 | 0.136 | 0.216 | 0.446 | 0.216 |
| **UD Radius Tb.Sp** | *****0.001** | 0.462 | ***0.010** | 0.361 | *****0.001** | 0.473 | ***0.027** | 0.316 | 0.209 | 0.183 | ***0.017** | 0.183 |
| **UD Radius Ct.vBMD** | ***0.039** | -0.296 | ***0.039** | -0.296 | ***0.0370** | -0.296 | ***0.049** | -0.283 | ****0.005** | -0.395 | 0.550 | -0.395 |
| **UD Radius Ct.Th** | ***0.016** | -0.342 | ****0.001** | -0.454 | ***0.019** | -0.331 | 0.073 | -0.258 | 0.117 | -0.227 | 0.253 | -0.227 |
| **UD Radius Ct.Po** | 0.097 | 0.240 | 0.587 | -0.080 | 0.554 | 0.086 | 0.324 | 0.144 | 0.101 | 0.237 | 0.366 | 0.237 |
|  |  |  |  |  |  |  |  |  |  |  |  |  |
| **Tibia Tt.vBMD** | ***0.011** | -0.361 | ****0.008** | -0.376 | ****0.003** | -0.416 | ***0.023** | -0.324 | 0.098 | -0.239 | 0.084 | -0.239 |
| **Tibia Tb.vBMD** | 0.117 | -0.227 | 0.069 | -0.262 | 0.058 | -0.270 | 0.070 | -0.261 | 0.495 | -0.100 | ***0.028** | -0.100 |
| **Tibia BV/TV** | 0.133 | -0.218 | 0.074 | -0.257 | 0.084 | -0.247 | 0.107 | -0.233 | 0.593 | -0.078 | ***0.039** | -0.078 |
| **Tibia Tb.N** | 0.134 | -0.217 | 0.176 | -0.197 | 0.186 | -0.190 | 0.099 | -0.239 | 0.738 | -0.049 | ***0.025** | -0.049 |
| **Tibia Tb.Th** | 0.657 | -0.065 | 0.322 | -0.144 | 0.324 | -0.143 | 0.343 | -0.138 | 0.291 | -0.154 | 0.462 | -0.154 |
| **Tibia Tb.Sp** | 0.167 | 0.200 | 0.204 | 0.184 | 0.185 | 0.191 | 0.114 | 0.229 | 0.681 | 0.060 | 0.054 | 0.060 |
| **Tibia Ct.vBMD** | ***0.036** | -0.300 | ***0.048** | -0.284 | ****0.001** | -0.441 | ***0.014** | -0.347 | ***0.022** | -0.328 | 0.072 | -0.328 |
| **Tibia Ct.Th** | ***0.015** | -0.346 | ***0.032** | -0.308 | ****0.002** | -0.423 | 0.148 | -0.210 | 0.086 | -0.248 | 0.308 | -0.248 |
| **Tibia Ct.Po** | 0.095 | 0.241 | ***0.043** | 0.290 | 0.404 | 0.121 | ****0.005** | 0.398 | 0.084 | 0.249 | 0.525 | 0.249 |

**Supplementary Table 2. r values and p values of the correlations between BMD, HRpQCT indices, TNF^+^ T-cells (frequency and MFI), Th17 cells (frequency and MFI), and PBMC *Tnf* and *Il17* transcripts levels in patients with normal bone density, osteopenia, or osteoporosis.**

Patients with Normal BMD (n =17)

|  | **TNF^+^ T cells (%)** | | **TNF^+^ T cells (MFI)** | | **PBMC Tnf mRNA** | | **Th17 cells (%)** | | **Th17 cells (MFI)** | | **PBMC Il17 mRNA** | |
| --- | --- | --- | --- | --- | --- | --- | --- | --- | --- | --- | --- | --- |
|  | p value | r value | p value | r value | p value | r value | p value | r value | p value | r value | p value | r value |
| **Spine BMD** | 0.099 | -0.413 | 0.250 | -0.295 | 0.227 | -0.309 | 0.272 | -0.282 | 0.620 | -0.130 | 0.300 | -0.267 |
| **1/3 Radius BMD** | 0.545 | 0.158 | 0.788 | 0.070 | 0.377 | -0.229 | 0.712 | -0.097 | 0.558 | 0.153 | 0.357 | -0.238 |
| **Total Hip BMD** | 0.309 | 0.271 | 0.334 | 0.258 | 0.902 | -0.034 | 0.931 | -0.024 | 0.187 | -0.348 | 0.588 | -0.146 |
| **FN BMD** | 0.553 | 0.160 | 0.547 | 0.163 | 0.730 | -0.094 | 0.677 | -0.113 | 0.616 | -0.136 | 0.855 | 0.050 |
|  |  |  |  |  |  |  |  |  |  |  |  |  |
| **1/3 Radius Tt.vBMD** | ***0.036** | 0.512 | 0.239 | 0.302 | 0.752 | -0.083 | 0.748 | -0.084 | 0.290 | 0.273 | 0.911 | -0.029 |
| **1/3 Radius Tb.vBMD** | 0.702 | -0.100 | 0.336 | -0.249 | 0.951 | 0.016 | 0.906 | 0.031 | 0.140 | 0.373 | 0.840 | -0.053 |
| **1/3 Radius BV/TV** | 0.448 | -0.197 | 0.234 | -0.305 | 0.926 | 0.024 | 0.981 | 0.006 | 0.416 | 0.211 | 0.985 | -0.005 |
| **1/3 Radius Tb.N** | 0.674 | -0.110 | 0.411 | -0.213 | 0.814 | 0.062 | 0.898 | 0.034 | 0.286 | 0.275 | 0.622 | 0.129 |
| **1/3 Radius Tb.Th** | 0.219 | -0.315 | 0.185 | -0.337 | 0.968 | 0.010 | 0.791 | -0.069 | 0.278 | 0.279 | 0.999 | 0.000 |
| **1/3 Radius Tb.Sp** | 0.832 | 0.056 | 0.541 | 0.159 | 0.544 | -0.158 | 0.706 | -0.099 | 0.286 | -0.275 | 0.788 | -0.070 |
| **1/3 Radius Ct.vBMD** | ***0.026** | 0.538 | 0.150 | 0.365 | 0.525 | -0.166 | 0.892 | 0.036 | 0.615 | 0.132 | 0.852 | 0.049 |
| **1/3 Radius Ct.Th** | 0.167 | 0.351 | 0.294 | 0.270 | 0.793 | -0.069 | 0.898 | -0.034 | 0.519 | 0.168 | 0.633 | -0.125 |
| **1/3 Radius Ct.Po** | 0.505 | -0.174 | 0.604 | -0.135 | 0.750 | 0.083 | 0.332 | -0.250 | ***0.026** | 0.539 | 0.847 | -0.051 |
|  |  |  |  |  |  |  |  |  |  |  |  |  |
| **UD Radius Tt.vBMD** | 0.303 | 0.265 | 0.623 | -0.129 | 0.331 | -0.251 | 0.991 | -0.003 | 0.847 | 0.051 | 0.659 | 0.115 |
| **UD Radius Tb.vBMD** | 0.708 | 0.098 | 0.592 | -0.140 | 0.154 | -0.362 | 0.657 | 0.116 | 0.540 | 0.160 | 0.672 | -0.111 |
| **UD Radius BV/TV** | 0.736 | 0.088 | 0.597 | -0.138 | 0.134 | -0.378 | 0.474 | 0.186 | 0.751 | 0.083 | 0.649 | -0.119 |
| **UD Radius Tb.N** | 0.957 | 0.014 | 0.479 | -0.184 | 0.223 | -0.312 | 0.601 | -0.137 | 0.454 | 0.195 | 0.976 | -0.008 |
| **UD Radius Tb.Th** | 0.588 | 0.141 | 0.955 | -0.015 | 0.798 | -0.067 | 0.367 | 0.233 | 0.976 | 0.008 | 0.444 | -0.199 |
| **UD Radius Tb.Sp** | 0.764 | -0.079 | 0.564 | 0.150 | 0.392 | 0.222 | 0.424 | 0.207 | 0.330 | -0.251 | 0.870 | -0.043 |
| **UD Radius Ct.vBMD** | 0.744 | 0.086 | 0.842 | 0.052 | 0.887 | 0.037 | 0.119 | -0.393 | 0.539 | -0.160 | 0.218 | 0.315 |
| **UD Radius Ct.Th** | 0.162 | 0.355 | 0.991 | -0.003 | 0.776 | -0.075 | 0.892 | 0.036 | 0.944 | 0.018 | 0.966 | 0.011 |
| **UD Radius Ct.Po** | 0.949 | -0.017 | 0.288 | -0.274 | 0.219 | -0.314 | 0.073 | 0.446 | 0.955 | 0.015 | 0.249 | -0.296 |
|  |  |  |  |  |  |  |  |  |  |  |  |  |
| **Tibia Tt.vBMD** | 0.406 | 0.216 | 0.991 | 0.003 | 0.748 | -0.084 | 0.878 | 0.040 | 0.798 | -0.067 | 0.560 | 0.152 |
| **Tibia Tb.vBMD** | 0.503 | 0.174 | 0.756 | 0.081 | 0.846 | -0.051 | 0.295 | 0.270 | 0.623 | 0.129 | 0.440 | -0.201 |
| **Tibia BV/TV** | 0.548 | 0.157 | 0.796 | 0.068 | 0.859 | -0.047 | 0.210 | 0.320 | 0.616 | 0.131 | 0.434 | -0.203 |
| **Tibia Tb.N** | 0.926 | -0.024 | 0.936 | -0.021 | 0.910 | -0.030 | 0.747 | -0.084 | 0.355 | 0.239 | 0.332 | -0.250 |
| **Tibia Tb.Th** | 0.816 | 0.061 | 1.000 | 0.000 | 0.670 | -0.111 | 0.479 | 0.184 | 0.527 | -0.165 | 0.450 | -0.196 |
| **Tibia Tb.Sp** | 0.774 | -0.075 | 0.908 | -0.030 | 0.980 | -0.006 | 0.888 | -0.037 | 0.328 | -0.253 | 0.445 | 0.199 |
| **Tibia Ct.vBMD** | 0.495 | 0.178 | 0.879 | 0.040 | 0.974 | -0.008 | 0.154 | -0.362 | 0.908 | 0.030 | 0.229 | 0.308 |
| **Tibia Ct.Th** | 0.358 | 0.238 | 0.968 | 0.011 | 0.617 | -0.131 | 0.823 | 0.059 | 0.361 | -0.236 | 0.499 | 0.176 |
| **Tibia Ct.Po** | 0.312 | 0.261 | 0.672 | 0.111 | 0.709 | -0.098 | 0.063 | 0.460 | 0.239 | 0.302 | 0.499 | -0.176 |

Patients with Osteopenia (n =18)

|  | **TNF^+^ T cells (%)** | | **TNF^+^ T cells (MFI)** | | **PBMC Tnf mRNA** | | **Th17 cells (%)** | | **Th17 cells (MFI)** | | **PBMC Il17 mRNA** | |
| --- | --- | --- | --- | --- | --- | --- | --- | --- | --- | --- | --- | --- |
|  | p value | r value | p value | r value | p value | r value | p value | r value | p value | r value | p value | r value |
| **Spine BMD** | 0.845 | 0.051 | 0.664 | 0.114 | 0.422 | -0.202 | 0.580 | 0.145 | 0.121 | 0.390 | 0.090 | -0.411 |
| **1/3 Radius BMD** | ***0.047** | -0.489 | 0.596 | -0.138 | 0.061 | -0.449 | ***0.050** | -0.483 | ***0.030** | -0.526 | ****0.001** | -0.694 |
| **Total Hip BMD** | 0.096 | -0.417 | 0.308 | -0.263 | ***0.031** | -0.509 | 0.850 | -0.050 | 0.412 | -0.213 | ***0.028** | -0.517 |
| **FN BMD** | 0.227 | -0.309 | 0.822 | -0.059 | 0.221 | -0.303 | 0.420 | -0.209 | 0.913 | -0.029 | ****0.003** | -0.651 |
|  |  |  |  |  |  |  |  |  |  |  |  |  |
| **1/3 Radius Tt.vBMD** | ***0.021** | -0.553 | 0.054 | -0.474 | ***0.038** | -0.492 | ***0.022** | -0.551 | ****0.001** | -0.721 | ***0.020** | -0.541 |
| **1/3 Radius Tb.vBMD** | 0.110 | 0.402 | 0.988 | -0.004 | ***0.012** | 0.577 | 0.716 | -0.095 | 0.689 | 0.105 | 0.320 | 0.249 |
| **1/3 Radius BV/TV** | 0.426 | -0.207 | 0.532 | 0.163 | 0.826 | 0.056 | 0.718 | 0.094 | ****0.007** | 0.630 | 0.773 | -0.073 |
| **1/3 Radius Tb.N** | 0.320 | -0.256 | 0.130 | -0.382 | 0.710 | 0.094 | 0.127 | -0.385 | 0.105 | -0.407 | 0.200 | -0.317 |
| **1/3 Radius Tb.Th** | ****0.009** | 0.616 | 0.098 | 0.415 | ****0.001** | 0.701 | 0.286 | 0.275 | ***0.036** | 0.512 | ***0.049** | 0.470 |
| **1/3 Radius Tb.Sp** | 0.298 | 0.268 | 0.058 | 0.469 | 0.791 | -0.067 | 0.199 | 0.328 | 0.133 | 0.379 | 0.445 | 0.192 |
| **1/3 Radius Ct.vBMD** | 0.132 | -0.381 | 0.142 | -0.371 | ***0.034** | -0.501 | ***0.049** | -0.483 | 0.093 | -0.420 | ***0.012** | -0.576 |
| **1/3 Radius Ct.Th** | ***0.021** | -0.554 | 0.335 | -0.249 | 0.053 | -0.463 | ***0.020** | -0.560 | ****0.007** | -0.628 | ****0.0036** | -0.649 |
| **1/3 Radius Ct.Po** | ***0.019** | 0.561 | 0.114 | 0.397 | ****0.004** | 0.639 | 0.112 | 0.400 | 0.070 | 0.451 | 0.176 | 0.334 |
|  |  |  |  |  |  |  |  |  |  |  |  |  |
| **UD Radius Tt.vBMD** | ***0.027** | -0.535 | 0.056 | -0.472 | 0.114 | -0.386 | 0.730 | -0.090 | 0.059 | -0.466 | 0.338 | -0.240 |
| **UD Radius Tb.vBMD** | ***0.048** | -0.485 | 0.139 | -0.374 | 0.070 | -0.437 | 0.113 | -0.399 | 0.223 | -0.312 | ***0.041** | -0.486 |
| **UD Radius BV/TV** | 0.104 | -0.408 | 0.429 | -0.206 | 0.134 | -0.367 | 0.216 | -0.316 | 0.545 | -0.158 | ***0.013** | -0.574 |
| **UD Radius Tb.N** | ***0.044** | -0.494 | ***0.047** | -0.488 | 0.088 | -0.413 | ***0.013** | -0.588 | 0.078 | -0.439 | 0.116 | -0.384 |
| **UD Radius Tb.Th** | 0.605 | -0.135 | 0.152 | 0.363 | 0.733 | -0.087 | ***0.033** | 0.519 | 0.189 | 0.335 | 0.070 | -0.436 |
| **UD Radius Tb.Sp** | ***0.037** | 0.509 | 0.057 | 0.470 | 0.117 | 0.383 | ***0.022** | 0.550 | 0.185 | 0.337 | 0.143 | 0.359 |
| **UD Radius Ct.vBMD** | 0.063 | -0.460 | 0.123 | -0.389 | 0.136 | -0.366 | 0.941 | -0.020 | ***0.028** | -0.533 | 0.293 | -0.262 |
| **UD Radius Ct.Th** | 0.072 | -0.447 | 0.247 | -0.297 | 0.240 | -0.292 | 0.944 | 0.019 | 0.078 | -0.439 | 0.476 | -0.179 |
| **UD Radius Ct.Po** | ****0.002** | 0.699 | 0.097 | 0.415 | 0.142 | 0.360 | 0.563 | 0.151 | 0.123 | 0.388 | 0.090 | 0.412 |
|  |  |  |  |  |  |  |  |  |  |  |  |  |
| **Tibia Tt.vBMD** | 0.121 | -0.391 | 0.153 | -0.363 | ***0.041** | -0.485 | 0.268 | -0.285 | ***0.029** | -0.529 | 0.207 | -0.312 |
| **Tibia Tb.vBMD** | 0.863 | -0.045 | 0.473 | -0.187 | 0.409 | -0.207 | ****0.001** | -0.723 | ***0.049** | -0.484 | 0.260 | -0.280 |
| **Tibia BV/TV** | 0.957 | -0.014 | 0.538 | -0.161 | 0.507 | -0.167 | ****0.003** | -0.669 | 0.057 | -0.470 | 0.302 | -0.257 |
| **Tibia Tb.N** | 0.821 | -0.059 | 0.317 | -0.258 | 0.865 | 0.043 | ****0.005** | -0.648 | 0.396 | -0.220 | 0.395 | -0.213 |
| **Tibia Tb.Th** | 0.596 | 0.139 | 0.824 | 0.058 | 0.586 | -0.138 | 0.452 | -0.196 | ***0.049** | -0.484 | 0.449 | 0.190 |
| **Tibia Tb.Sp** | 0.794 | 0.068 | 0.382 | 0.226 | 0.953 | -0.015 | ****0.006** | 0.640 | 0.472 | 0.187 | 0.317 | 0.250 |
| **Tibia Ct.vBMD** | 0.256 | -0.292 | 0.487 | -0.181 | ***0.044** | -0.479 | 0.998 | 0.001 | 0.195 | -0.331 | ***0.012** | -0.578 |
| **Tibia Ct.Th** | 0.122 | -0.390 | 0.427 | -0.206 | 0.074 | -0.431 | 0.732 | 0.090 | 0.273 | -0.282 | 0.912 | 0.028 |
| **Tibia Ct.Po** | 0.651 | 0.118 | 0.589 | 0.141 | 0.692 | 0.100 | 0.512 | 0.171 | 0.657 | 0.116 | ****0.003** | 0.660 |

Patients with Osteoporosis (n=15)

|  | **TNF^+^ T cells (%)** | | **TNF^+^ T cells (MFI)** | | **PBMC Tnf mRNA** | | **Th17 cells (%)** | | **Th17 cells (MFI)** | | **PBMC Il17 mRNA** | |
| --- | --- | --- | --- | --- | --- | --- | --- | --- | --- | --- | --- | --- |
|  | p value | r value | p value | r value | p value | r value | p value | r value | p value | r value | p value | r value |
| **Spine BMD** | 0.587 | -0.153 | 0.749 | 0.090 | ****0.001** | -0.760 | 0.200 | -0.351 | **0.006 | -0.669 | **0.003** | -0.715 |
| **1/3 Radius BMD** | ***0.012** | -0.631 | 0.067 | -0.484 | 0.141 | -0.399 | 0.212 | -0.342 | 0.489 | -0.194 | 0.281 | -0.298 |
| **Total Hip BMD** | 0.123 | -0.416 | 0.781 | -0.078 | ***0.042** | -0.531 | 0.366 | -0.251 | 0.172 | -0.372 | **0.036** | -0.545 |
| **FN BMD** | 0.129 | -0.410 | 0.478 | -0.199 | 0.165 | -0.377 | 0.590 | -0.151 | 0.730 | -0.097 | 0.295 | -0.290 |
|  |  |  |  |  |  |  |  |  |  |  |  |  |
| **1/3 Radius Tt.vBMD** | 0.197 | -0.353 | 0.079 | -0.467 | 0.141 | -0.399 | 0.233 | -0.328 | 0.284 | -0.296 | 0.265 | -0.308 |
| **1/3 Radius Tb.vBMD** | 0.612 | -0.143 | 0.209 | -0.344 | 0.465 | -0.204 | 0.480 | 0.198 | 0.904 | -0.034 | 0.274 | -0.302 |
| **1/3 Radius BV/TV** | 0.797 | -0.073 | 0.275 | -0.301 | 0.809 | -0.068 | 0.276 | 0.301 | 0.770 | 0.083 | 0.544 | -0.170 |
| **1/3 Radius Tb.N** | 0.635 | -0.134 | 0.487 | -0.195 | 0.225 | -0.333 | 0.815 | -0.066 | 0.496 | -0.191 | 0.146 | -0.394 |
| **1/3 Radius Tb.Th** | 0.237 | -0.325 | 0.154 | -0.387 | 0.567 | -0.161 | 0.568 | 0.160 | 0.712 | 0.104 | 0.651 | -0.127 |
| **1/3 Radius Tb.Sp** | 0.654 | 0.126 | 0.457 | 0.208 | 0.140 | 0.399 | 0.616 | 0.141 | 0.233 | 0.328 | ***0.05** | 0.517 |
| **1/3 Radius Ct.vBMD** | 0.363 | -0.253 | 0.753 | -0.089 | 0.149 | -0.391 | 0.316 | -0.278 | 0.844 | 0.056 | 0.887 | -0.040 |
| **1/3 Radius Ct.Th** | 0.073 | -0.476 | 0.111 | -0.428 | 0.119 | -0.420 | 0.137 | -0.402 | 0.311 | -0.281 | 0.166 | -0.377 |
| **1/3 Radius Ct.Po** | 0.358 | 0.256 | 0.768 | -0.083 | 0.090 | 0.453 | ****0.007** | 0.665 | 0.364 | 0.253 | 0.591 | 0.151 |
|  |  |  |  |  |  |  |  |  |  |  |  |  |
| **UD Radius Tt.vBMD** | ***0.022** | -0.584 | ***0.021** | -0.590 | 0.119 | -0.420 | 0.191 | -0.357 | 0.713 | 0.104 | 0.480 | -0.198 |
| **UD Radius Tb.vBMD** | ****0.008** | -0.656 | 0.154 | -0.387 | ***0.012** | -0.629 | 0.309 | -0.282 | 0.733 | 0.096 | 0.209 | -0.344 |
| **UD Radius BV/TV** | ****0.009** | -0.647 | 0.203 | -0.349 | ***0.018** | -0.600 | 0.234 | -0.327 | 0.598 | 0.148 | 0.310 | -0.281 |
| **UD Radius Tb.N** | ****0.003** | -0.715 | 0.138 | -0.402 | ***0.012** | -0.627 | 0.497 | -0.190 | 0.922 | -0.028 | 0.116 | -0.424 |
| **UD Radius Tb.Th** | 0.312 | -0.280 | 0.369 | -0.250 | 0.656 | -0.126 | 0.692 | -0.112 | 0.090 | 0.453 | 0.356 | 0.257 |
| **UD Radius Tb.Sp** | ****0.003** | 0.710 | 0.217 | 0.339 | ***0.012** | 0.632 | 0.590 | 0.151 | 0.673 | 0.119 | 0.070 | 0.480 |
| **UD Radius Ct.vBMD** | 0.914 | -0.030 | 0.413 | -0.228 | 0.538 | -0.173 | 0.297 | -0.289 | 0.421 | -0.224 | 0.775 | -0.081 |
| **UD Radius Ct.Th** | ***0.022** | -0.585 | ***0.014** | -0.619 | 0.356 | -0.256 | 0.137 | -0.402 | 0.604 | 0.146 | 0.673 | -0.119 |
| **UD Radius Ct.Po** | 0.974 | -0.009 | 0.731 | -0.097 | 0.428 | 0.221 | 0.762 | 0.086 | 0.062 | 0.492 | 0.148 | 0.393 |
|  |  |  |  |  |  |  |  |  |  |  |  |  |
| **Tibia Tt.vBMD** | ****0.004** | -0.702 | 0.105 | -0.435 | 0.091 | -0.451 | 0.079 | -0.467 | 0.855 | 0.052 | 0.170 | -0.374 |
| **Tibia Tb.vBMD** | ***0.042** | -0.531 | 0.190 | -0.358 | 0.259 | -0.311 | 0.222 | -0.335 | 0.470 | 0.202 | 0.357 | -0.256 |
| **Tibia BV/TV** | ***0.028** | -0.564 | 0.159 | -0.383 | 0.295 | -0.289 | 0.221 | -0.336 | 0.377 | 0.246 | 0.438 | -0.217 |
| **Tibia Tb.N** | 0.617 | -0.141 | 0.998 | -0.001 | 0.341 | -0.265 | 0.829 | -0.061 | 0.635 | 0.134 | 0.325 | -0.273 |
| **Tibia Tb.Th** | ***0.014** | -0.619 | 0.057 | -0.500 | 0.358 | -0.256 | 0.115 | -0.424 | 0.712 | 0.104 | 0.587 | -0.153 |
| **Tibia Tb.Sp** | 0.688 | 0.113 | 0.958 | -0.015 | 0.364 | 0.253 | 0.960 | 0.014 | 0.556 | -0.165 | 0.484 | 0.196 |
| **Tibia Ct.vBMD** | 0.188 | -0.360 | 0.652 | -0.127 | 0.064 | -0.489 | 0.194 | -0.355 | 0.135 | -0.404 | 0.107 | -0.433 |
| **Tibia Ct.Th** | ****0.003** | -0.717 | 0.195 | -0.355 | 0.081 | -0.465 | 0.151 | -0.390 | 0.999 | 0.000 | 0.178 | -0.368 |
| **Tibia Ct.Po** | 0.921 | -0.028 | 0.655 | 0.126 | 0.813 | -0.067 | 0.206 | 0.346 | 0.725 | 0.099 | 0.567 | -0.161 |

**Supplementary Table 3.** Bacterial Species associated to spine BMD.

| Species | p-value | q-value |
| --- | --- | --- |
| *Blautia.sp900555025* | 0.0068 | 0.862 |
| *Blautia.pseudococcoides* | 0.0093 | 0.862 |
| *Facklamia.hominis* | 0.0093 | 0.862 |
| *Falseniella.ignava* | 0.0093 | 0.862 |
| *Peptoniphilus_C.pacaensis* | 0.0093 | 0.862 |
| *Streptococcus.cristatus* | 0.0093 | 0.862 |
| *Blautia.sp900120295* | 0.0094 | 0.862 |
| *Clostridium_AQ.innocuum* | 0.0102 | 0.862 |
| *Olsenella_F.sp001189515* | 0.0129 | 0.862 |
| *Dorea_B.phocaeensis* | 0.0146 | 0.862 |
| *Pseudobutyricicoccus.sp003477405* | 0.016 | 0.862 |
| *Blautia.sp000432195* | 0.0161 | 0.862 |
| *Blautia.sp001304935* | 0.0177 | 0.862 |
| *Akkermansia.muciniphila_B* | 0.0202 | 0.862 |
| *Raoultibacter.timonensis* | 0.0212 | 0.862 |
| *Parabacteroides.goldsteinii* | 0.0218 | 0.862 |
| *Blautia_A.caecimuris* | 0.0234 | 0.862 |
| *Bacteroides.intestinalis* | 0.025 | 0.862 |
| *Alistipes.timonensis* | 0.0255 | 0.862 |
| *Raoultibacter.massiliensis* | 0.0258 | 0.862 |
| *Granulicatella_u_s* | 0.027 | 0.862 |
| *Mediterraneibacter.lactaris* | 0.028 | 0.862 |
| *Akkermansia_u_s* | 0.029 | 0.862 |
| *Bacteroides.eggerthii* | 0.03 | 0.862 |
| *Bacteroides.uniformis* | 0.0325 | 0.862 |
| *Amedibacillus.dolichus* | 0.0332 | 0.862 |
| *Lactococcus.cremoris* | 0.034 | 0.862 |
| *Corynebacterium.durum* | 0.0343 | 0.862 |
| *Adlercreutzia.muris* | 0.035 | 0.862 |
| *Blautia.coccoides* | 0.0353 | 0.862 |
| *Anaerostipes.sp900066705* | 0.0367 | 0.862 |
| *Collinsella_u_s* | 0.0407 | 0.862 |
| *Anaerotruncus.rubiinfantis* | 0.0433 | 0.862 |
| *Christensenella.massiliensis* | 0.045 | 0.862 |
| *Anaerostipes.hadrus* | 0.048 | 0.862 |
| *Neobittarella.massiliensis* | 0.0497 | 0.862 |

**Supplementary Table 4.** Bacterial Species associated to 1/3 radius BMD.

| Species | p-value | q-value |
| --- | --- | --- |
| *Akkermansia_u_s* | 0.0076 | 0.9926 |
| *Phocea.massiliensis* | 0.0076 | 0.9926 |
| *Granulicatella_u_s* | 0.0114 | 0.9926 |
| *Klebsiella.pneumoniae* | 0.0146 | 0.9926 |
| *Raoultibacter.massiliensis* | 0.0168 | 0.9926 |
| *Blautia.sp900555025* | 0.018 | 0.9926 |
| *Collinsella_u_s* | 0.0204 | 0.9926 |
| *Blautia.pseudococcoides* | 0.0293 | 0.9926 |
| *Facklamia.hominis* | 0.0293 | 0.9926 |
| *Falseniella.ignava* | 0.0293 | 0.9926 |
| *Peptoniphilus_C.pacaensis* | 0.0293 | 0.9926 |
| *Streptococcus.cristatus* | 0.0293 | 0.9926 |
| *Eubacterium_G.sp000434315* | 0.0328 | 0.9926 |
| *Lactobacillus.delbrueckii* | 0.034 | 0.9926 |
| *Lactococcus.cremoris* | 0.034 | 0.9926 |
| *Blautia.sp000432195* | 0.0347 | 0.9926 |
| *Thomasclavelia.saccharogumia* | 0.0377 | 0.9926 |
| *Akkermansia.muciniphila_B* | 0.0387 | 0.9926 |
| *Phocaeicola.plebeius* | 0.044 | 0.9926 |
| *Paraprevotella.xylaniphila* | 0.0443 | 0.9926 |
| *Blautia_A.caecimuris* | 0.0447 | 0.9926 |
| *Parabacteroides.goldsteinii* | 0.045 | 0.9926 |
| *Blautia.coccoides* | 0.0457 | 0.9926 |
| *Blautia.sp900120295* | 0.0457 | 0.9926 |
| *Blautia.sp001304935* | 0.0463 | 0.9926 |
| *Bacteroides.bouchesdurhonensis* | 0.0483 | 0.9926 |
| *Bacteroides.propionicigenes* | 0.0483 | 0.9926 |
| *Bacteroides.sp012113595* | 0.0483 | 0.9926 |
| *Bacteroides.sp014750685* | 0.0483 | 0.9926 |
| *Blautia_A.sp018918125* | 0.0483 | 0.9926 |
| *Blautia_A.sp018919065* | 0.0483 | 0.9926 |
| *Parabacteroides.sp014287585* | 0.0483 | 0.9926 |
| *Paraprevotella.sp003477995* | 0.0483 | 0.9926 |
| *Prevotella.rara* | 0.0483 | 0.9926 |
| *Eisenbergiella.porci* | 0.0497 | 0.9926 |

**Supplementary Table 5.** Beta values of the mediation analysis indices shown in Fig. 5b.

| Exposure | Bifidobacterium Longum | |
| --- | --- | --- |
| Mediator | PBMC Il17 mRNA  (Relative expression) | PBMC Tnf mRNA  (Relative expression) |
|  |  |  |
| beta-value a | 0.161 | 0.281 |
| beta--value b  1/3 Radius BMD | -0.018 | -0.012 |
| beta--value b  1/3 Radius Ct.vBMD | -2.613 | -5.247 |
| beta--value b  Spine BMD | -0.031 | -0.025 |
| beta--value ACME  1/3 Radius BMD | -0.027 | -0.031 |
| beta value ACME  1/3 Radius Ct vBMD | -3.966 | -13.889 |
| beta--value ACME  Spine BMD | -0.047 | -0.067 |
| beta--value ADE  1/3 Radius BMD | -0.018 | -0.014 |
| beta value ADE  1/3 Radius Ct vBMD | -8.137 | 1.786 |
| beta--value ADE  Spine BMD | -0.037 | -0.016 |

**Supplementary Table 6.** Sensitivity indices for the mediation analysis shown in Fig. 5b.

Rho_at_min_abs_ACME, R2star_d_thresh, and Rtilde_d_thresh.

| Exposure | Mediator | Outcome | Rho at min abs ACME | R2star d  threshold | R2 tilde d  threshold |
| --- | --- | --- | --- | --- | --- |
| *Bifidobacterium Longum* | PBMC *Il17* mRNA  (Relative expression) | 1/3 radius BMD | -0.3 | 0.09 | 0.029 |
|  | PBMC *Il17* mRNA  (Relative expression) | 1/3 radius  Ct. vBMD | -0.1 | 0.01 | 0.006 |
|  | PBMC *Il17* mRNA  (Relative expression) | Spine BMD | -0.3 | 0.09 | 0.051 |
|  | PBMC *Tnf* mRNA  (Relative expression) | 1/3 radius BMD | -0.3 | 0.09 | 0.021 |
|  | PBMC *Tnf* mRNA  (Relative expression) | 1/3 radius  Ct. vBMD | -0.3 | 0.09 | 0.033 |
|  | PBMC *Tnf* mRNA  (Relative expression) | Spine BMD | -0.3 | 0.09 | 0.036 |


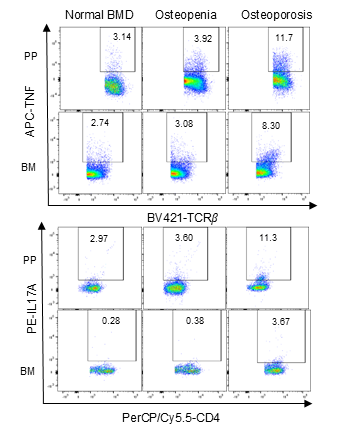

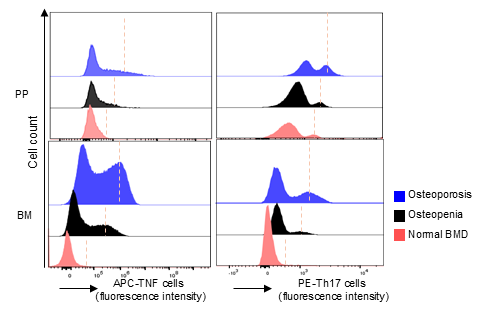


a

b

c


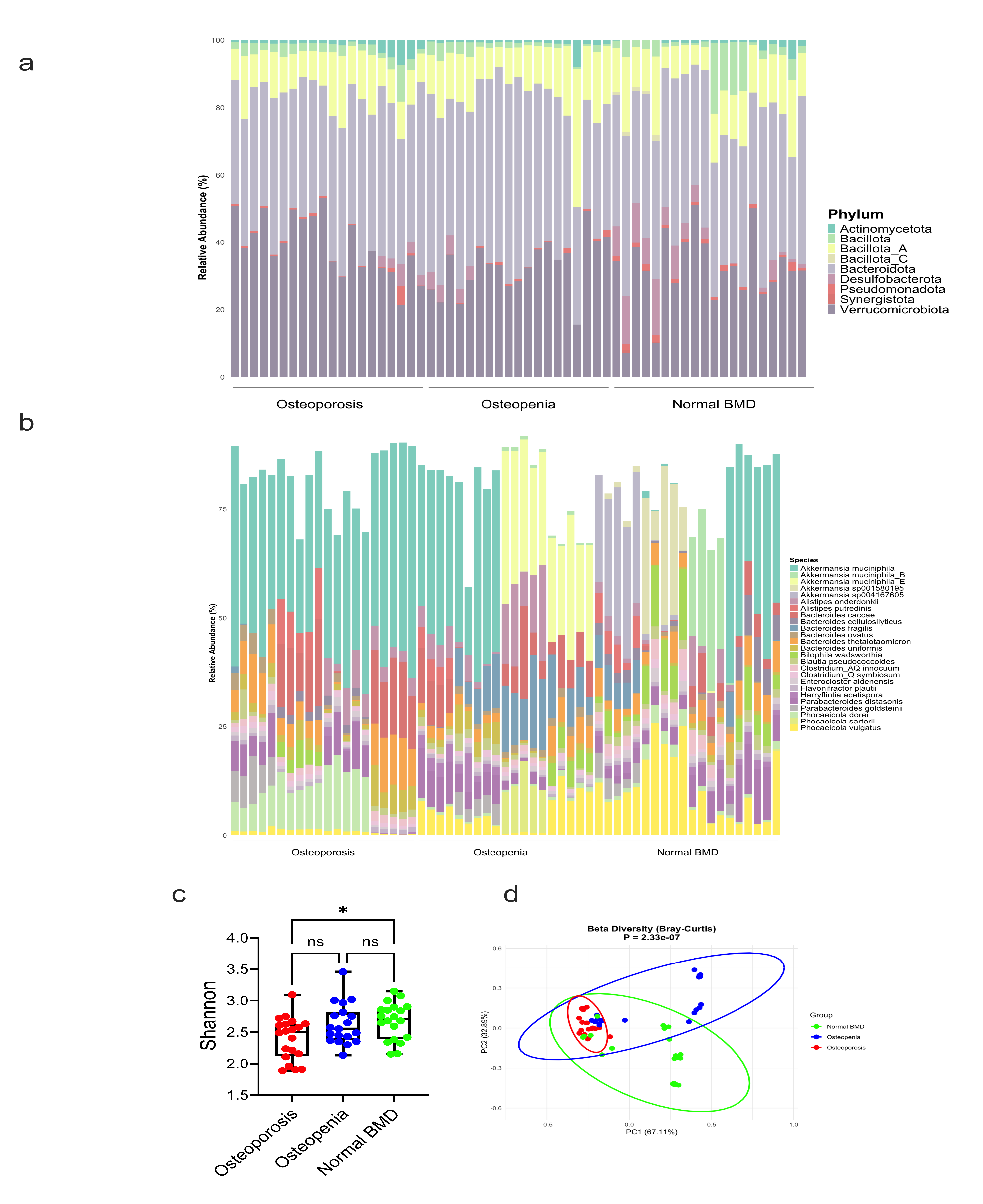


**Supplementary Fig. 1. a Gating strategy and representative flow cytometry data. a** Gating strategy used to identify TNF^+^ T-cells and Th17 cells in peripheral blood mononuclear cells (PBMCs). Following red blood cells lysis, single cell suspensions were prepared from PBMCs and stained with antibodies to the indicated antigens and live/dead cell dye. Gated regions are numbered from R1 to R5. The figure shows one representative gating of flow cytometric plots. **b** Percentage of Peyer’s patches (PP) and bone marrow (BM) TNF^+^ T cells and Th17 cells in one representative patient per group. **c** Mean fluorescence intensity (MFI) of PP and BM TNF^+^ T cells and Th17 cells in one representative patient per group.

**Supplementary Fig. 2 | Analysis of** **stool microbiome of germ-free mice colonized with stool microbiome from PHPT patients with osteoporosis, osteopenia, or normal bone density. a** Relative phylum level abundance. **b** Stacked bars representing the distribution of the 25 most abundant species. **c** Bacterial species alpha diversity analysis conducted by calculating the Shannon diversity index. Kruskal-Wallis test was used to test for significant differences between groups. The box shows the median and Q1-Q3 interquartile range, the bars show the minimum and maximum values. **d** Principal coordination analysis (PCoA) of bacterial species. PCoA plots were based on the Bray-Curtis distance metrics. PERMANOVA multivariate analysis was used to test for significant differences between groups. n = 4-5 recipient mice per human donor. Kruskal-Wallis test was used to test for significant differences between groups. * = p<0.05 and **** = p<0.0001. ns = not significant.


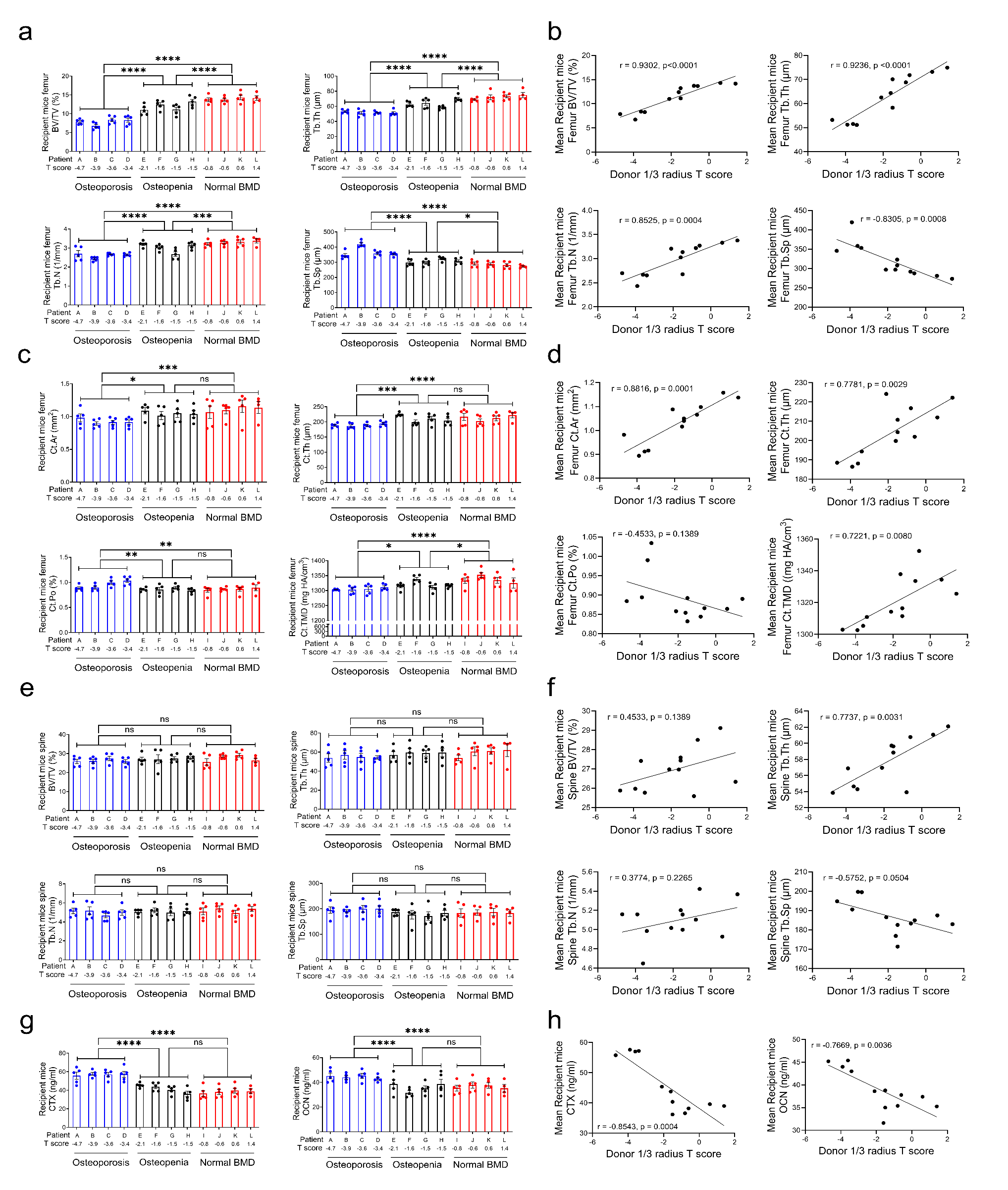


**Supplementary Fig. 3 |** **Femoral and spinal (L4) indices of trabecular and cortical bone structure of germ-free mice with microbiome from PHPT patients with 1/3 radius T scores in the range of osteoporosis, osteopenia, or normal BMD.** The measured indices were trabecular bone volume/total volume ratio (BV/TV), trabecular thickness (Tb.Th), trabecular number (Tb.N), trabecular separation (Tb.Sp), cortical area (Ct.Ar), cortical thickness (Ct.Th), cortical porosity (Ct.Po), cortical tissue mineral density (Ct.TMD), C-terminal telopeptide (CTX), which is marker of bone resorption, and osteocalcin (OCN), which is a marker of bone formation**.** In panels (**a, c, e, g**), each dot represents a recipient mouse (independent biological replicate). Each letter represents a patient. Mice were divided into 3 groups based on the 1/3 radius T score of the human donors and the groups were compared by one-way ANOVA and post hoc tests applying Bonferroni’s correction for multiple comparisons. Data are shown as mean + SEM. n = 4-5 recipient mice for each human donor. In panels (**b, d, f, h**) the 1/3 radius T score of each human donor was plotted against the mean of the values measured in the 4-5 recipient mice with the microbiome from the same donor. r and p values were calculated using Pearson correlations. The curves show simple linear regression lines. * = p<0.05, ** = p<0.01, *** = p<0.001, and **** = p<0.0001. ns = not significant.


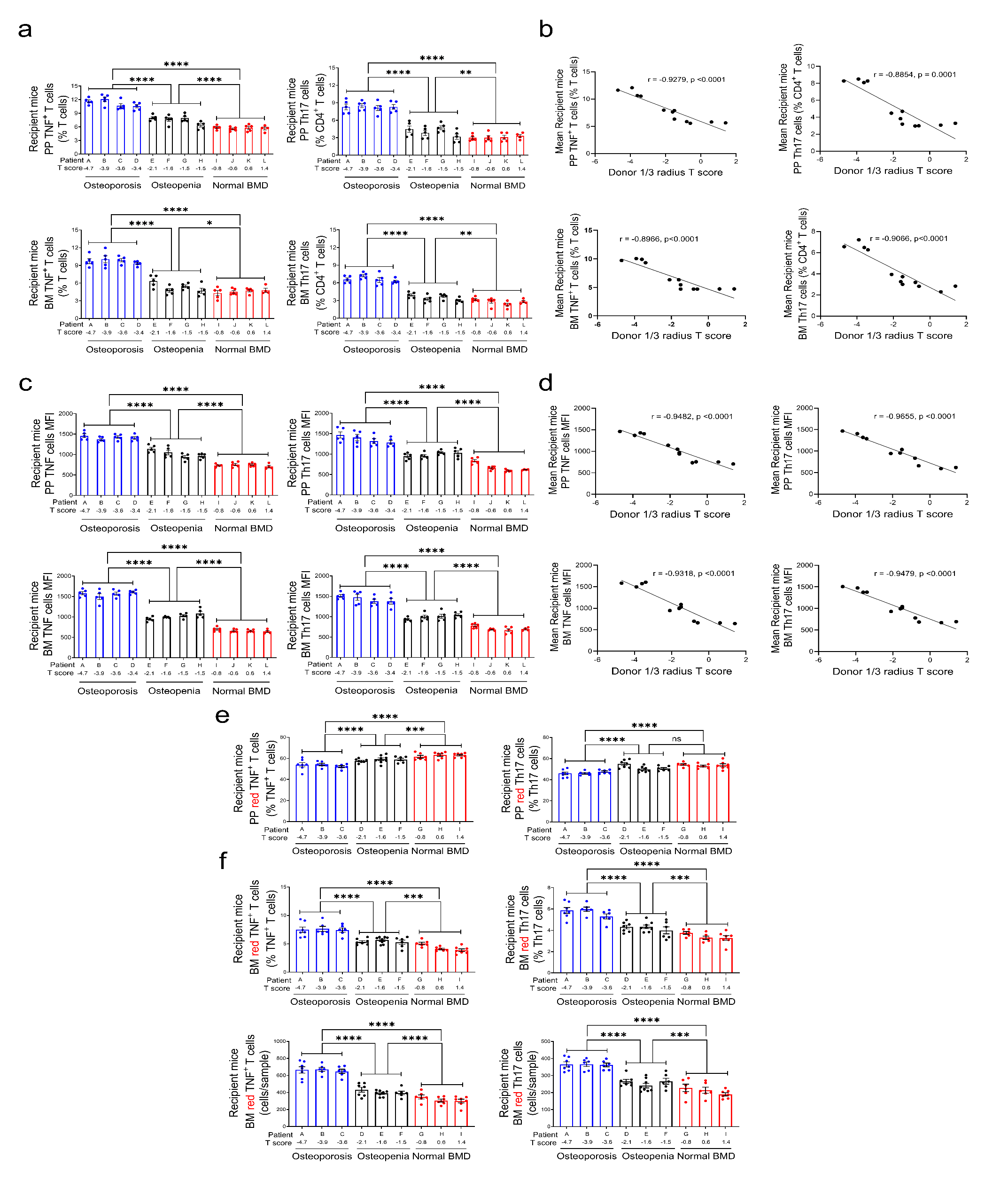


**Supplementary Fig. 4 | Frequency and MFI of PP and BM of TNF^+^ T-cells and Th17 cells in WT germ-free mice, and frequency of red fluorescent tagged TNF^+^ T-cells and Th17 cells in germ-free Kaede mice with microbiome from PHPT patients with 1/3 radius T scores in the range of osteoporosis, osteopenia, or normal BMD.** In panels (**a, c, e, f**), each dot represents a recipient mouse (independent biological replicate). Each letter represents a patient. Mice were divided in 3 groups based on the 1/3 radius T score of the human donors and the groups were compared by one-way ANOVA and post hoc tests applying Bonferroni’s correction for multiple comparisons. In panels (**b, d**), the 1/3 radius T score of each human donor was plotted against the mean of the values measured in the recipient mice with the microbiome from the same donor. r and p values were calculated using Pearson correlations. The curves show simple linear regression lines. All data were normally distributed. Data are shown as mean + SEM. n = 4-5 WT mice and 6-8 Kaede mice per each human donor. * = p<0.05, ** = p<0.01, *** = p< 0.001, and **** = p<0.0001.

**Supplemental Figure 4. Analysis of stool microbiome of PHPT patients. (**A**)** Stacked bars representing the distribution of the most abundant Phila. **(**B**)** Stacked bars representing the distribution of the most abundant species. **(**C) Bacterial species alpha diversity analysis. (D) Principal coordination analysis (PCoA) of bacterial species. Alpha diversity analysis was conducted by calculating the Shannon diversity index by gender. The p-values were generated from the Wilcoxon-rank-sum test. The box shows the median and Q1-Q3 interquartile range, the bars show the minimum and maximum values. PCoA plots were based on the Bray-Curtis distance metrics. The p-values were generated from the PERMANOVA test. n = 9-14 ns = not significant.


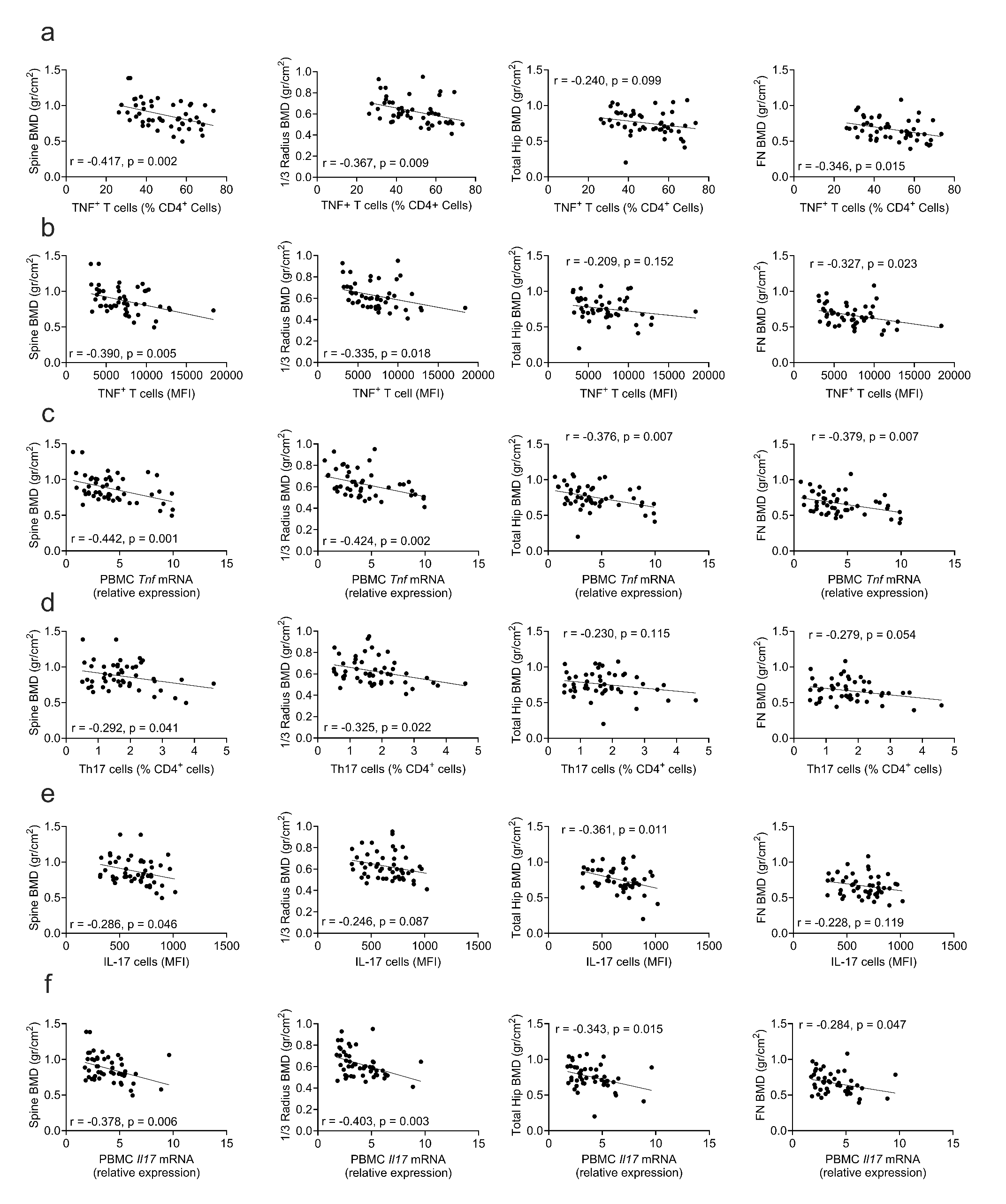


**Supplementary Fig. 5 |** **Correlations between BMD and TNF^+^ T-cells (frequency and MFI), Th17 cells (frequency and MFI), and PBMC *Tnf* and *Il17* transcripts levels.** Graphic representations of **a** BMD versus TNF^+^ T-cells %. **b** BMD versus TNF^+^ T-cells MFI. **c** BMD versus PBMC *Tnf* mRNA level. **d** BMD versus Th17 T-cells %. **e** BMD versus Th17 cells MFI. **f** BMD versus PBMC *Il17a* mRNA level. r and p values have been calculated using Pearson correlations. The curves show simple linear regression lines. n = 49 for total hip and FN BMD. n = 50 for all other variables. DXA of the proximal femur was not done in 1 patient due to hip replacements.


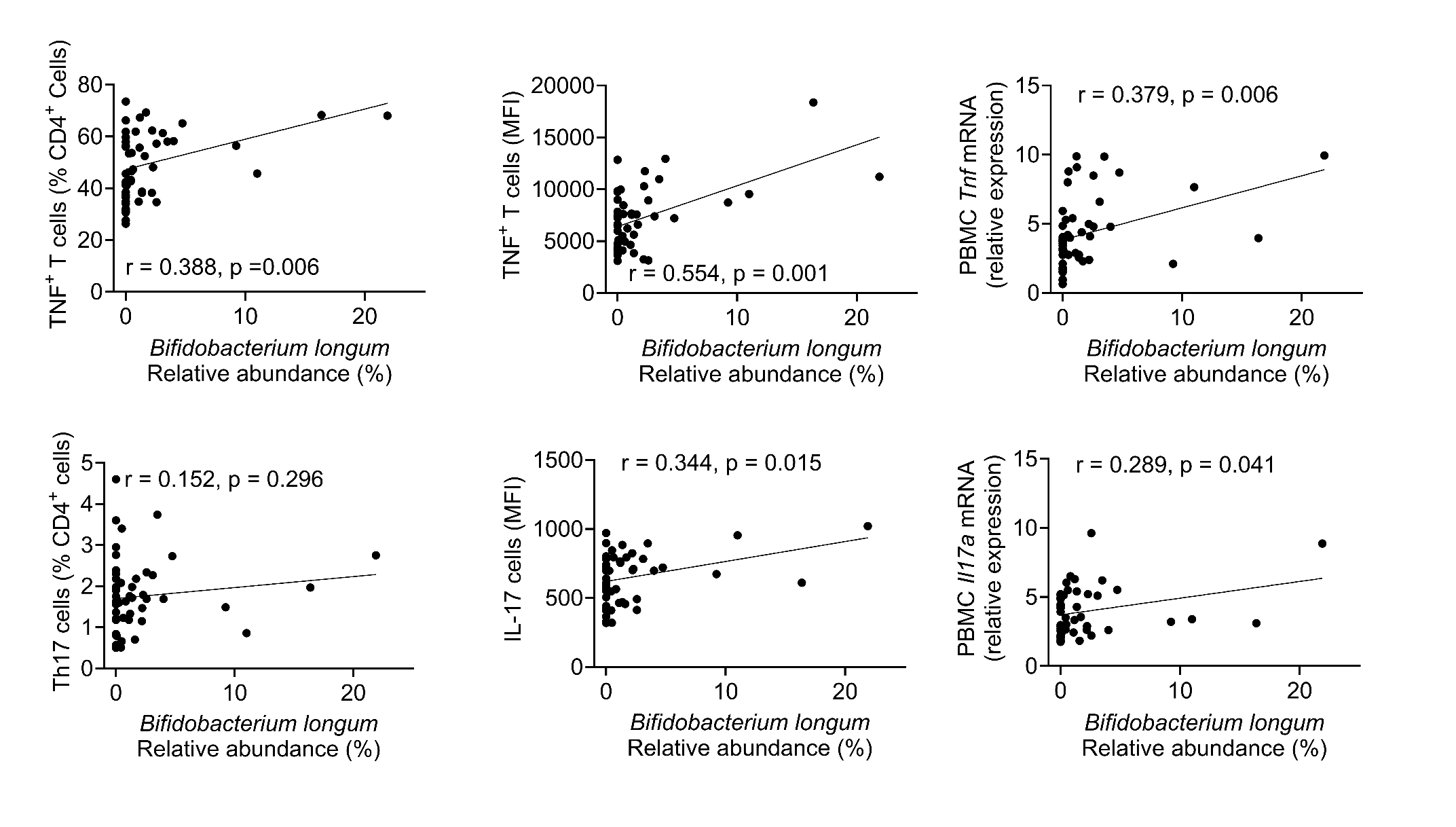


**Supplementary Fig. 6 |** **Correlations between the relative abundance of *Bifidobacterium longum* and frequency and MFI of TNF^+^ T cells and Th17 cells, and PBMC *Tnf* and *Il17* transcripts levels**. r and p values were calculated using Spearman correlations. The curves show simple linear regression lines. n = 50.


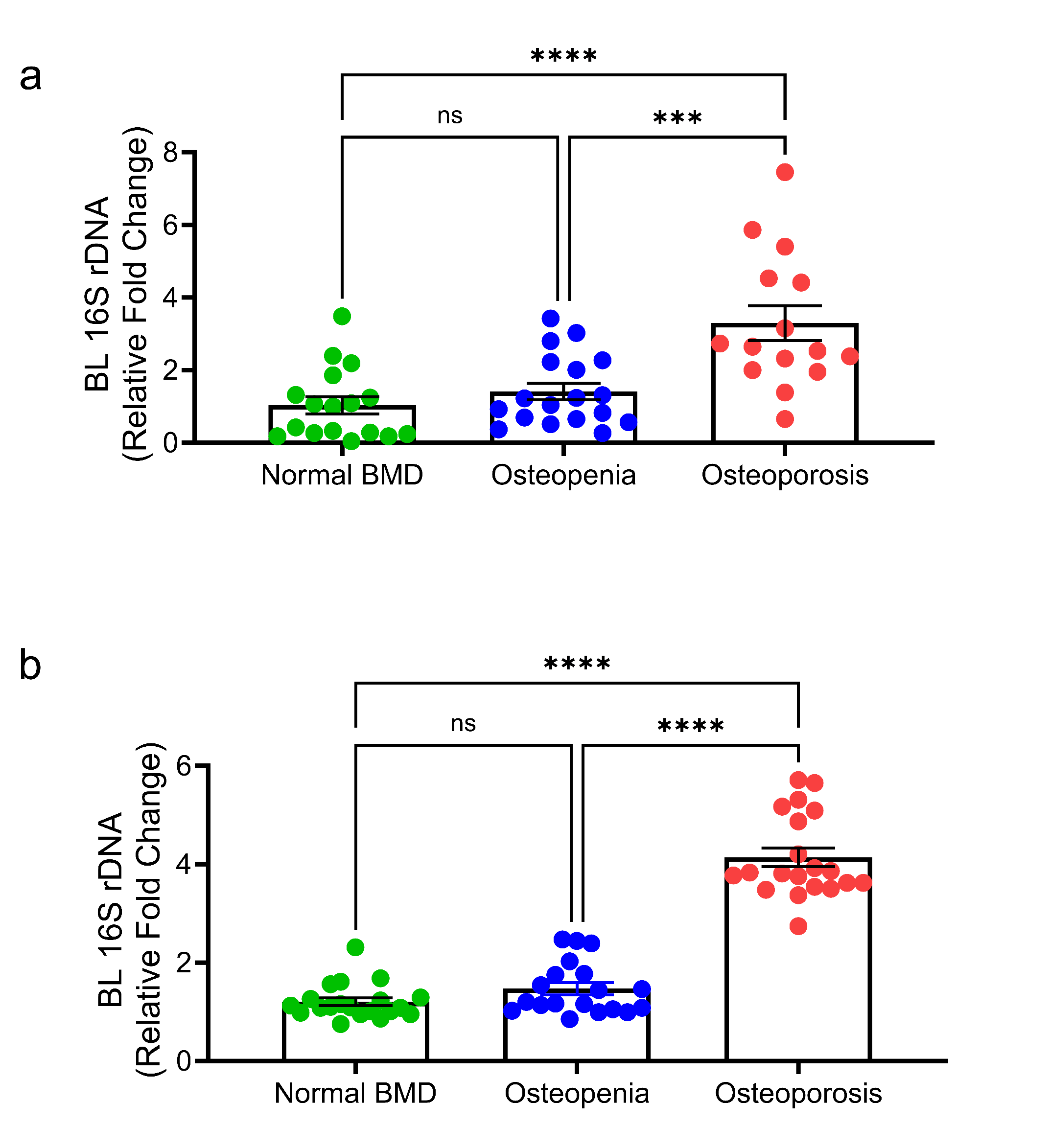


**Supplementary Fig. 7. a**. Levels of *Bifidobacterium longum* (BL)16S rDNA in stool samples from hyperparathyroidism patients with osteoporosis osteopenia or normal bone density. **b**. Levels of Bifidobacterium longum (BL)16S rDNA in stool samples from germ-free mice colonized with microbiome originating from hyperparathyroidism patients with osteoporosis, osteopenia or normal bone density. After fecal DNA extraction, the BL16S rDNA gene expression was quantified by qPCR using specific primers. *** = p<0.001. **** = p<0.0001. ns = not significant.

**Supplementary Fig. 8 |** **Monocolonization of germ-free mice with *Bifidobacterium longum* (BL) or daily gavages of BL in conventional mice increased stool BL but not SFB 16S rDNA levels.** **a** BL 16S rDNA levels in stool samples from germ-free mice monocolonized with BL. **b** SFB 16S rDNA levels in stool samples from germ-free mice monocolonized with BL. **c** BL 16S rDNA levels in stool samples from conventional mice gavaged daily with BL from 16- to 20-weeks of age. In all experiments BL or SFB 16S rDNA gene expression was quantified by qPCR using specific primers after fecal DNA extraction. ** = p<0.01.


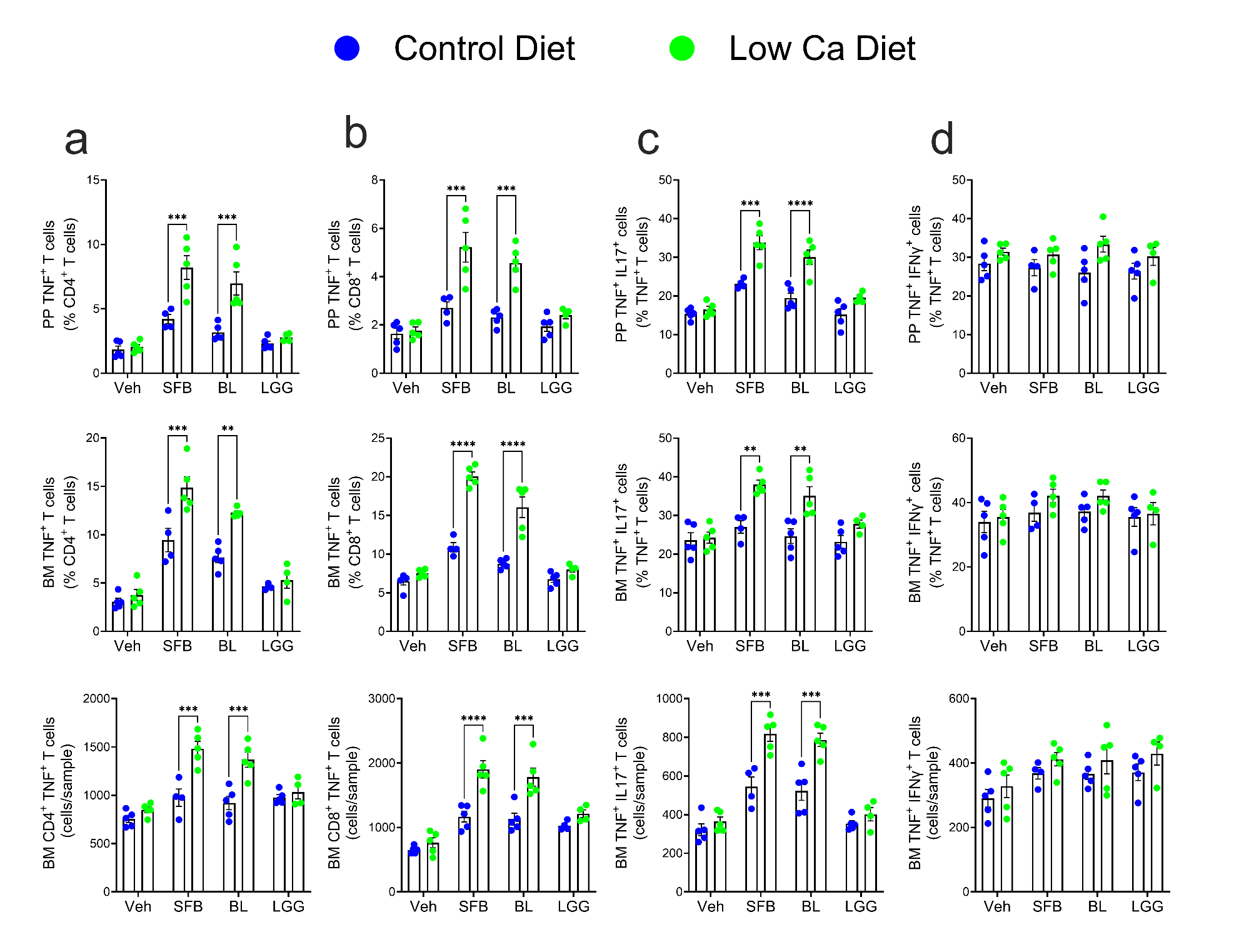


**Supplementary Fig. 9 |** **Monocolonization of germ-free mice with *BifidobacteriumlLongum* (BL) is sufficient for low calcium diet to increase the frequency of PP and BM CD4^+^TNF^+^ T cells, CD8^+^TNF^+^ T cells, TNF^+^IL-17^+^ T cells, but not TNF^+^IFNγ^+^ T cells.** In these experiments WT germ-free mice monocolonized with SFB were used as positive controls. Germ-free mice monocolonized with vehicle or LGG were used as negative controls. **a** CD4^+^TNF^+^ T cells. **b** CD8^+^TNF^+^ T cells. **c** TNF^+^IL-17^+^ T cells. **d** TNF^+^IFNγ^+^ T cells. n = 4-7 mice/group. Data were expressed as Mean + SEM and were normally distributed according to the Shapiro-Wilk normality test. Data were analyzed by two-way ANOVA and post hoc tests applying the Bonferroni correction for multiple comparisons. ** = p<0.01, *** = p< 0.001 and **** = p<0.0001. Non-significant comparisons not shown.


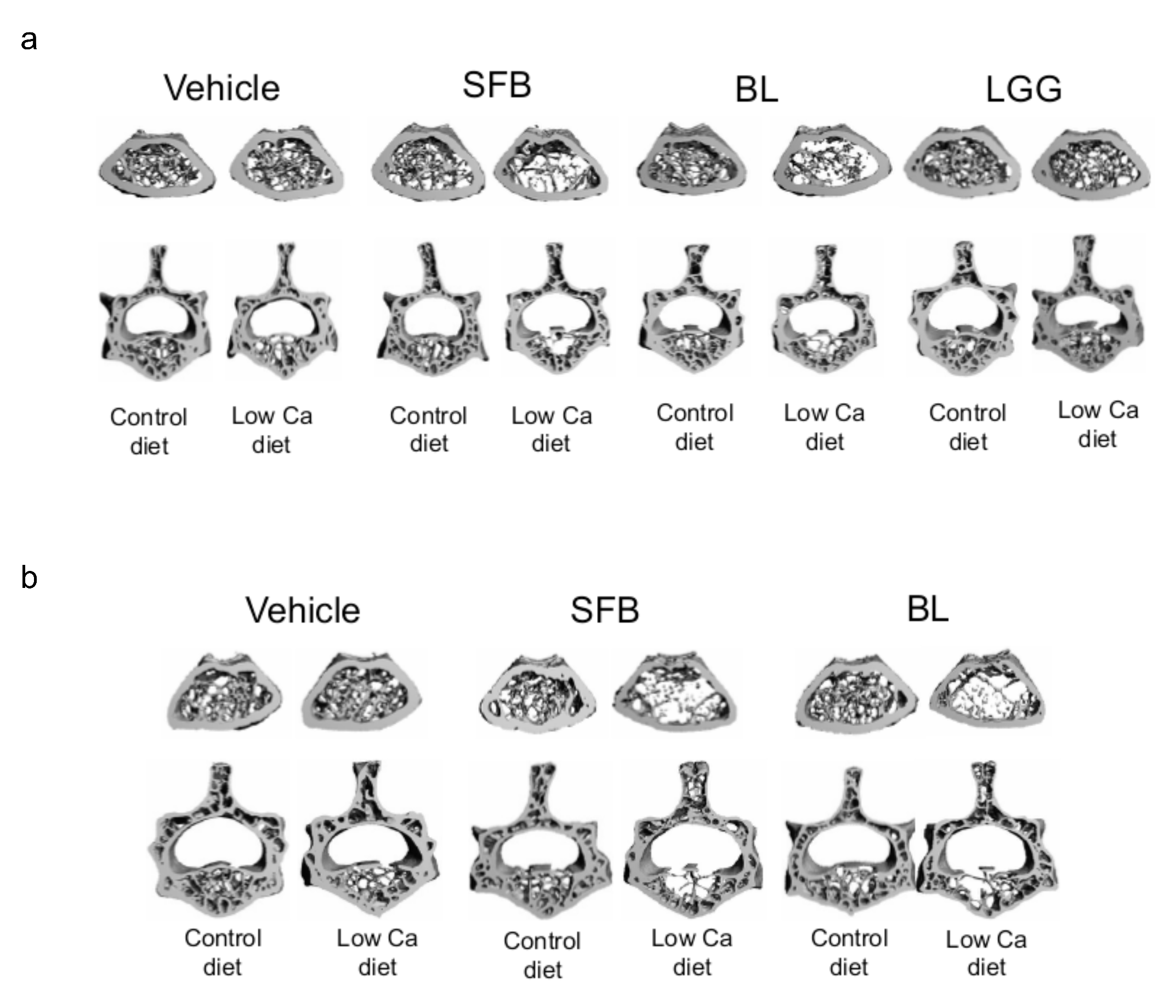


**Supplementary Fig. 10 |** **Images of representative 3-dimensional μCT reconstructions of examined femurs and spine.** **a** Monocolonization of germ-free mice with *Bifidobacterium Longum* (BL) induced cortical and trabecular bone loss in the femur and the spine. **b** Daily oral supplementation of conventionally raised mice with BL induced femoral and spinal bone loss.


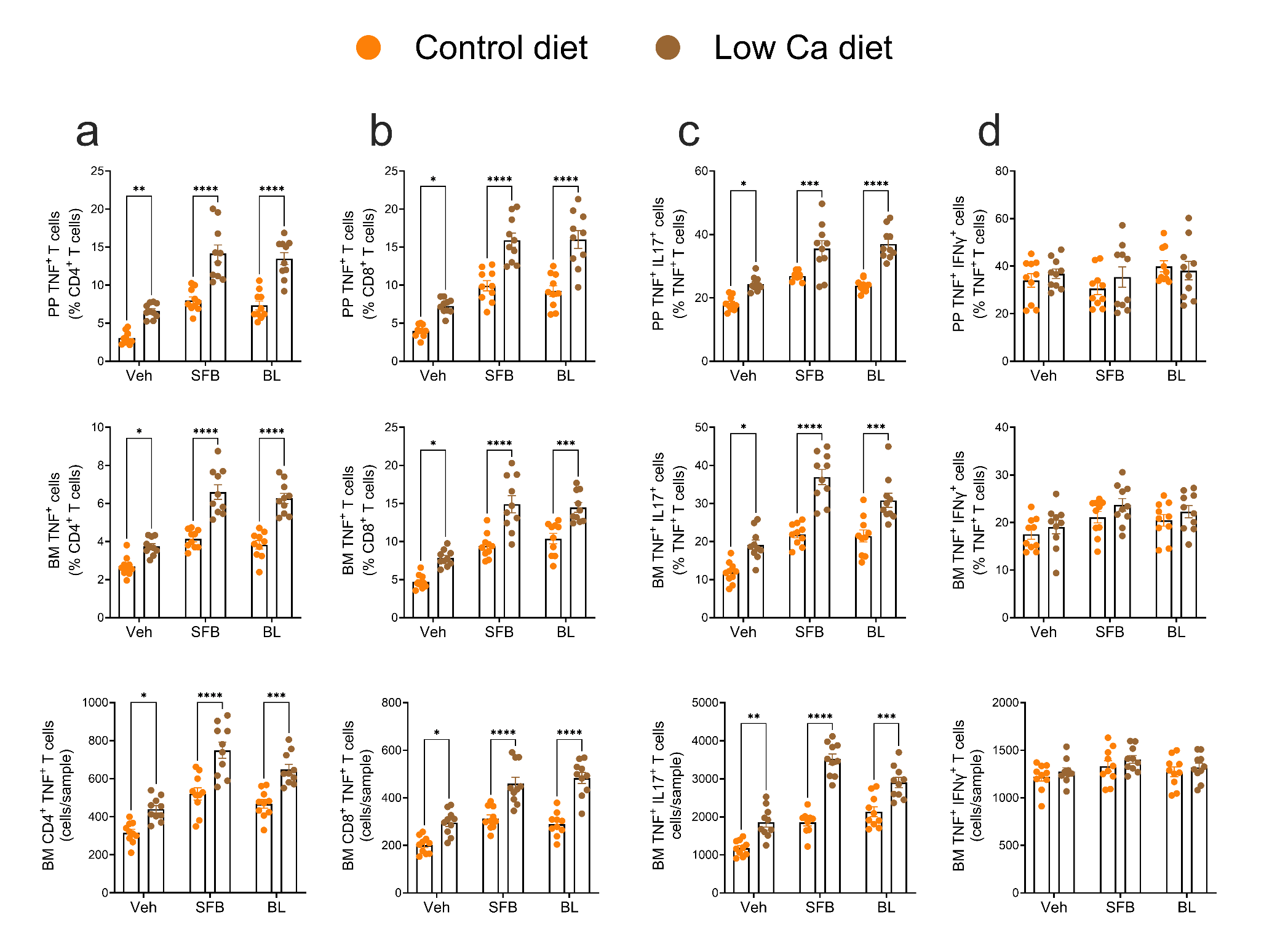


**Supplementary Fig. 11 |** **Daily oral supplementation of conventionally raised mice with *Bifidobacterium longum* (BL) is sufficient for a low calcium diet to increase the frequency of PP and BM CD4^+^TNF^+^ T cells, CD8^+^TNF^+^ T cells, TNF^+^IL-17^+^ T cells, but not TNF^+^IFNγ^+^ T cells.** In these experiments, WT germ-free mice monocolonized with SFB were used as positive controls. Germ-free mice monocolonized with vehicle or LGG were used as negative controls. **a** CD4^+^TNF^+^ T cells. **b** CD8^+^TNF^+^ T cells. **c** TNF^+^IL-17^+^ T cells. **d** TNF^+^IFNγ^+^ T cells. n = 4-7 mice/group. Data were expressed as Mean + SEM and were normally distributed according to the Shapiro-Wilk normality test. Data were analyzed by two-way ANOVA and post hoc tests applying the Bonferroni correction for multiple comparisons. * = p<0.05, ** = p<0.01, *** = p < 0.001, and **** = p<0.0001. Non-significant comparisons not shown.
